# Polysaccharide-Based Coatings as Drug Delivery Systems

**DOI:** 10.3390/pharmaceutics15092227

**Published:** 2023-08-29

**Authors:** Anita Ioana Visan, Rodica Cristescu

**Affiliations:** National Institute for Lasers, Plasma and Radiation Physics, 409 Atomistilor Street, 077125 Magurele, Ilfov, Romania

**Keywords:** drug delivery systems, polysaccharides, coatings, clinical applications

## Abstract

Therapeutic polysaccharide-based coatings have recently emerged as versatile strategies to transform a conventional medical implant into a drug delivery system. However, the translation of these polysaccharide-based coatings into the clinic as drug delivery systems still requires a deeper understanding of their drug degradation/release profiles. This claim is supported by little or no data. In this review paper, a comprehensive description of the benefits and challenges generated by the polysaccharide-based coatings is provided. Moreover, the latest advances made towards the application of the most important representative coatings based on polysaccharide types for drug delivery are debated. Furthermore, suggestions/recommendations for future research to speed up the transition of polysaccharide-based drug delivery systems from the laboratory testing to clinical applications are given.

## 1. Introduction: Background, Significance, and Justification

At present, the most commonly used drug delivery systems (e.g., inorganic nanoparticles, liposomes, micelles, and hydrogels) are important tools for delivering therapeutics to the patient. Moreover, multidisciplinary scientists around the world are exploring coatings of various materials as new drug delivery tools. Moreover, many surface coatings based on polysaccharides have been proposed to impart antimicrobial properties to implantable materials, mainly metals and polymers. The solution of medical surface functionalization with antimicrobial agents exists as an alternative approach to traditional drug dosage forms. This delivery system can be utilized for both systemic and topical purposes and can be administered through different routes, such as oral, buccal, sublingual, ocular, and transdermal, depending on the intended use. Therefore, the effective coating design demands extensive knowledge of the pharmacological properties of drugs and polysaccharides, as well as the appropriate selection of manufacturing processes.

**Figure 1:**
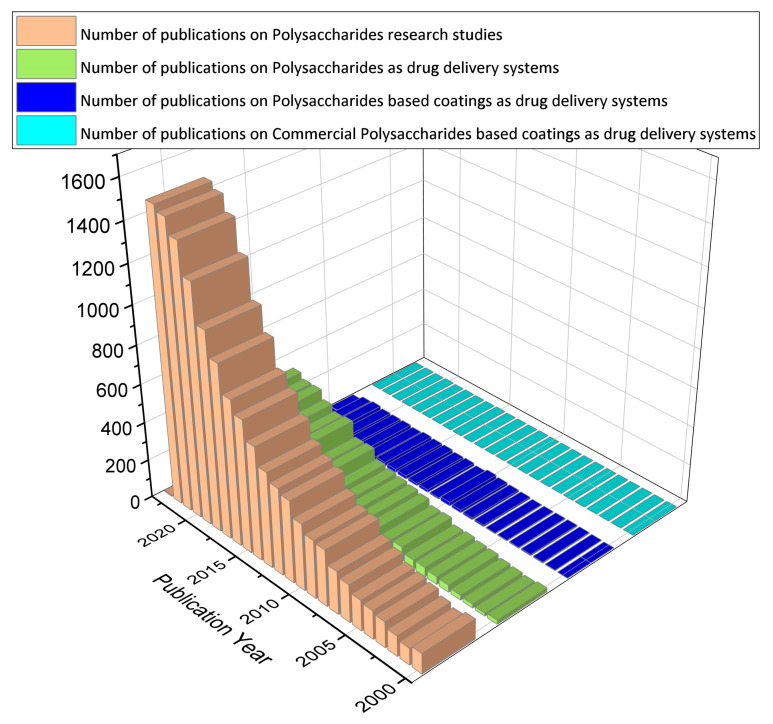
A digital survey based on the topic of polysaccharides, performed for the period 2000–2022, using data from Web of Science [5].

In this review article, we aim at collecting and discussing the polysaccharide-based coatings as drug delivery systems. Thin films made from polysaccharides are generally flexible layers of polymer that may or may not contain plasticizers [1]. As the functionalization of the surface coatings is provided with a thin and flexible layer of polysaccharides containing the antimicrobial agent, this method may prove to be less intrusive and more acceptable to patients [2]. In addition, coatings allow for targeting in sensitive areas that would otherwise not be possible only with liquid formulations or tablets [3]. On the other hand, this approach could reduce dosing frequency, eliminate drug side effects, and improve drug efficacy [4]. To date, most published studies (Figure 1) have used polysaccharides and derivatives in different drug formulations (those that use cleavable chemical bonds to connect polysaccharides and drugs, those that self-assemble to form particles with drugs inside, and those that encapsulate drugs in gel-like substances) (92%). However, the disadvantages of low mechanical resistance and uncontrolled hydration rate limit the use of polysaccharides in some drug delivery strategies (5%). Future extensions could include polysaccharides as coatings (2%). Currently, the polysaccharides enclosed in commercial formulations are only a few, being mainly used for research purposes (1%).

Comparative studies related to this area [6,7,8,9,10,11,12,13] have confirmed that it is imperative to conduct additional research and development in order to fully utilize the capabilities of polysaccharide-based coatings in the medical industry. The present review comprehensively evaluates 170 carefully selected publications from Web of Science and Science Direct databases, spanning the period between 2000 and 2022. We have included systematic reviews, research articles, and book chapters based on their relevance to the topic, research methodology, research results, and the year of publication.

## 2. Polysaccharide Administration Routes in Drug Delivery Systems

Polysaccharides offer versatile administration routes in drug delivery systems. They can be orally, parenterally, or topically administered, providing targeted delivery to specific tissues or cells. Concretely:

(i.) Oral administration: Polysaccharides can be formulated into nanoparticles/microparticles or films for oral drug delivery [7]. These particles can protect the drug from degradation in the gastrointestinal tract and enhance its absorption. Polysaccharide particles can adhere to mucosal surfaces, facilitating drug transport through the mucosal epithelium and improving bioavailability [7].
(ii.) Parenteral administration: Polysaccharide-based delivery systems can be administered via injection, allowing for direct delivery into the bloodstream [7]. This route enables rapid drug distribution and targeted delivery to specific tissues. Polysaccharide nanoparticles can circulate in the bloodstream for extended periods, avoiding rapid clearance by the reticuloendothelial system [7]. This prolonged circulation can enhance drug efficacy and reduce the frequency of administration.
(iii.) Topical administration: Polysaccharides can be incorporated into creams, gels, or patches for topical drugs [7]. When applied to the skin, polysaccharide-based formulations can provide sustained release of drugs, improving their therapeutic effect. The mucoadhesive properties of certain polysaccharides can enhance drug retention at the site of application, prolonging drug release and absorption [7].
(iv.) Targeted delivery: Polysaccharides offer the advantage of tissue-specific targeting in drug delivery [9]. By modifying the surface of polysaccharide particles or conjugating targeting ligands, drugs can be delivered to specific tissues or cells of interest. This targeted delivery approach improves drug efficacy and reduces off-target effects.
(v.) Combination with other delivery systems: Polysaccharides can be combined with other drug delivery systems, such as liposomes or nanoparticles, to enhance their absorption properties and functionality [7]. This combination can provide synergistic effects, such as improved drug stability, controlled release, and enhanced targeting capabilities.

Polysaccharides’ biocompatibility, biodegradability, and natural origin make them attractive for use in drug delivery, offering advantages in terms of safety, stability, and controlled release. The combination of polysaccharides and other delivery systems further expands their potential applications in drug delivery. Continued research and development in this field are likely to unlock new opportunities for polysaccharides in drug administration routes.

### Polysaccharide-Based Coatings Administration Routes in Drug Delivery Systems

Polysaccharide thin films can be also formulated for various routes of administration (including oral, transdermal, buccal, sublingual, ocular routes, mucoadhesive, transmucosal, and implants) and offer advantages such as controlled drug release and convenience of administration. Polysaccharide thin films considerations include the anatomical and physiological constraints of the target site, drug properties, and selection of suitable polysaccharides [11].

They serve different purposes such as quick drug absorption in the gastrointestinal cavity or delivering drugs at the site of administration. Coatings are ideal for drugs with high mucosal permeability, and buccal and sublingual delivery is common with them. Ophthalmic coatings are usually used to treat diseases of the anterior segment. Orodispersible films readily dissolve in the oral cavity and are commonly known as soluble films [14]. Fast-dissolving oral films have ultra-thin dimensions and dissolve within a minute in the oral cavity. Buccal adhesive films directly deliver drugs via the buccal mucosa for systemic circulation after their absorption. Wafers consist of paper-thin polymeric films used as carriers for pharmaceutical agents and do not require water for drug absorption [12]. Polysaccharide coatings are also used for surface modification of medical devices, especially for prosthetic applications in orthopedics and dentistry, for controlled drug release.

**Figure 2:**
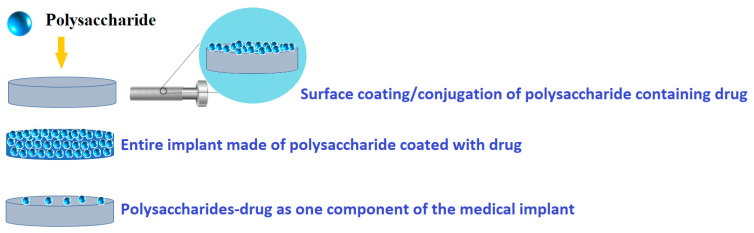
Common design possibilities for polysaccharide-based coatings as drug delivery systems.

In this review, the focus is on polysaccharide-based coatings for drug delivery, with special attention to metallic medical implants. Thus, there are possible different modalities of polysaccharide-based coatings as drug delivery systems (Figure 2). Therefore, using various deposition techniques, polysaccharides can be directly loaded onto a medical surface, while drugs and/or therapeutic agents can be loaded with polysaccharides using an overcoating, undercoating, reservoir coating, or coating with biodegradable polysaccharides.

Thin films can be formulated for drug delivery, using two approaches: directly loading drugs onto prefabricated polysaccharide thin films or preparing thin films using drugs as building blocks. There are several techniques available for loading drugs into polysaccharide thin films, which depend on the drug characteristics, the intended site of action, and the desired delivery outcome [6]. Moreover, polysaccharide thin films for drug delivery must be made either by directly loading drugs onto prefabricated films or using drugs as building blocks to ensure control over drug release [15]. The first approach is directly loading drugs onto prefabricated polysaccharide thin films. In this method, preformed thin films are used as carriers for loading drugs. The drugs can be incorporated into the films through physical adsorption, electrostatic interactions, or chemical bonding [16]. The release of the drug from the thin film can be controlled by factors such as film thickness, drug loading concentration, and the properties of the film material [6]. Another approach involves preparing thin films using drugs as building blocks. In this method, the drugs themselves are used as components to construct the thin films [16]. The drugs can be chemically modified or combined with other materials to form the thin films. This approach allows for precise control over the drug release kinetics and can be tailored to specific drug delivery requirements [17].

Ongoing research and development in this field aim to optimize the formulation and manufacturing processes of thin films for drug delivery, with the potential to improve patient outcomes and enhance drug delivery efficiency. Thus, the use of polysaccharide thin films in drug delivery provides an alternative to conventional dosage forms and offers convenience and effectiveness [17]. Concretely, several approaches have been investigated for the administration of polysaccharides in the form of coatings for drug delivery:

(i) Coating of the drug core: In this approach, the drug is coated with a polysaccharide material that can be broken down by the acid-mediated cleavage. This allows for the controlled release of the drug at the desired site [7].
(ii) Embedding of the drug in a biodegradable matrix: Polysaccharides can be used to form a matrix in which the drug is embedded. The matrix slowly degrades, releasing the drug over time [18].
(iii) Formulation of drug-saccharide conjugates (prodrugs): Polysaccharides can be chemically modified to form conjugates with drugs, resulting prodrugs. These prodrugs can be designed to release the active drug molecules, improving drug targeting and reducing systemic side effects [19].

The drug delivery systems have been sorted taking in account the involved physical or chemical mechanisms [20].

The physical process involves the breakdown of a polymer matrix which regulates the speed of drug release, as well as the movement of the drug through the polymer layer and the osmotic pressure that causes the drug to be released [21]. One benefit of utilizing physical mechanisms in drug delivery is the ability to adjust the kinetics of drug release by modifying factors, such as the thickness of polysaccharide membrane, type of used polysaccharide, and surface area of drug delivery system [22]. On the other hand, chemical mechanisms involve breaking covalent bonds, such as the bond between a drug and polymer, through chemical or enzymatic degradation [8]. In order to effectively manage these chemical mechanisms, the drug needs to undergo a chemical modification process for it to be grafted onto the polysaccharide. Because of the difficulties in synthesis, physical mechanisms have been employed more frequently than the chemical ones. The release of the drug from the polysaccharide system is greatly influenced by the degradation speed of used polysaccharide. As biodegradable polysaccharides are decomposed within the drug system, the drugs that were loaded are gradually released from the matrix, ensuring a consistent and efficient therapy. When producing polysaccharide-based coatings, the goal is to obtain stable, thin, and homogeneous functionalized surfaces.

**Figure 3:**
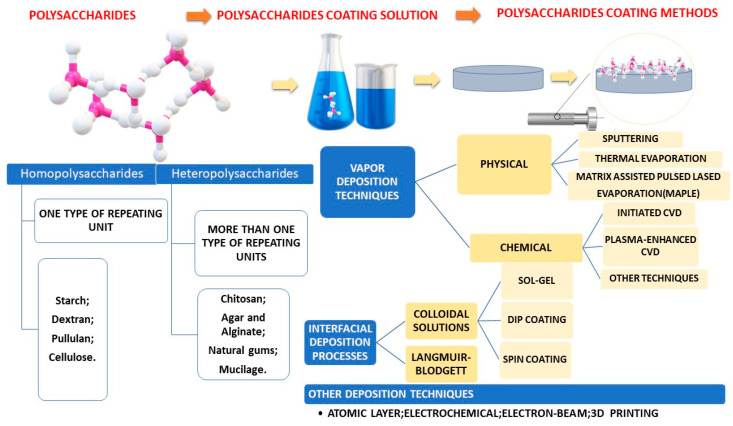
Schematic representation of most common polysaccharide-based coating fabrication methods.

Fabrication methods were centralized in Figure 3.

Thus, it is fully justified the need of a deeper study of these polysaccharide-based coatings as drug delivery systems for their clinical application in medical field, the advantages, and challenges of the design as coatings being represented in Table 1.

Coatings entirely made from polysaccharide–drug component are often brittle and fragile. To counteract this, a plasticizer could be introduced into the film-forming solution that enables the formation of an elastic structure. The plasticizer penetrates the molecular chains of polysaccharide, thus increasing cohesion. Polyols (such as polyethylene glycol, sorbitol, and glycerol), sugars (such as honey, and glucose), and lipids (such as phospholipids, and monoglycerides) are the most frequently utilized plasticizers. Proper selection of a plasticizer for polysaccharide-based coatings is important due to the fact that the coatings can undergo significant changes in their physicochemical properties [30].

In order to obtain a continuous structure of polysaccharide coatings, various polymers of biological origin were investigated. This was found to be necessary to incorporate into the polysaccharide hydrocolloid matrix (hydrophilic in nature) various types of oils and fats (e.g., acetylated monoglycerides, triglycerides) to improve its water vapor barrier properties [31].

The issue pertains to the degradation behavior of polysaccharides in drug delivery in form of coatings could be related to the polysaccharide chemistry, their broad and/or mixed molecular weights, making it difficult to precisely define the delivery vehicle. In drug delivery systems, the particle size distribution of the carrier is impacted by the molecular weight of polysaccharides. However, controlling the molecular weight of these polysaccharides can significantly increase their preparation costs. Moreover, often polysaccharide-based drug delivery platforms require the slow enzymatic degradation of biopolymers, and there are numerous polysaccharides that cannot dissolve in most organic solvents, which also restricts the options for their chemical modification [7]. Polysaccharides used as coatings may have limited applications due to their low drug loading capacity, which can be a problem when administering a less potent drug at a high dose [32]. Achieving an accurate amount of drug in each unit dose of the film can be a challenging task, and failure to do so can lead to therapeutic failure or nonreproducible effects [33]. This paper provides a comprehensive review of the latest developments in polysaccharide-based coatings for drug delivery systems. First, we examine the essential characteristics of an effective drug delivery system and highlight how coatings offer numerous benefits in this regard. Then, we discuss several prominent examples of polysaccharide-based coatings, as well as various methods proposed for their characterization. We also explore the commercialization challenges associated with these coatings and provide a thorough summary of the research conducted in this field. Finally, we offer conclusions and prospects for the future of this exciting area of study.

## 3. Overview on Polysaccharide-Based Drug Delivery Systems

### 3.1. Polysaccharide-Based Drug Delivery Systems Characteristics

The scientific community is continuously interested in polysaccharide-based drug delivery systems because of their exceptional properties, such as biocompatibility, solubility, and potential for modification. Additionally, several polysaccharides have innate bioactivity, which makes them promising for use in drug delivery systems. Concretely, polysaccharides are a diverse class of polymeric biomaterials of natural origin that are formed through glycosidic linkages of monosaccharides [30]. Polysaccharides can have either a linear or branched structure, depending on the type of monosaccharide unit they contain. They also have various reactive groups, such as hydroxyl, amino, and carboxylic acid groups, making them capable of being chemically modified [34].

In addition, the weight of polysaccharide molecules can range from hundreds to thousands of Daltons, which adds even more diversity [35]. Herein, next, we succinctly describe the following characteristics/merits of polysaccharides [36,37]:

(i) Biocompatibility and Biodegradability: Contrary to many synthetic polymers, polysaccharides have very low (if any) toxicity levels [38]. It is worth noting that many polysaccharides can be broken down by enzymes, resulting in the release of their monomer or oligomer building blocks. This process allows for the recycling of these building blocks for various purposes, such as storage, structural support, and cell signaling [39]. It is worth mentioning that other polysaccharides are particularly susceptible to degradation by lysosomal enzymes after endocytosis, including esterases, glycosidases and proteases [40]. Thus, enzymatic degradation offers a proper way to release therapeutics from polysaccharide-based carrier systems [41].
(ii) Bioactivity: Numerous polysaccharides possess inherent bioactivity, notably mucoadhesion, anti-inflammatory, and antimicrobial properties. Mucoadhesion refers to the bond between a substance and mucosal layer, such as in the gastrointestinal tract, nasal pathway, or airway [42]. Some polysaccharides such as chitosan have antimicrobial properties, while others such as heparin are known to help in reducing inflammation.
(iii) Solubility: Sometimes, solubility can be changed by adjusting the monomer (basic units of polymers) structure. For instance, it is possible to alter the solubility of chitosan in acidic conditions. Polysaccharides have functional groups (hydroxyl and amine groups) along their backbone, which usually result in high solubility in water [43]. For instance, when the degree of deacetylation is higher, it results in more protonated free amino groups along the polysaccharide backbone. This leads to improved solubility [44].
(iv) Ease of Modification: Polysaccharides can be easily modified. Glucose-based polysaccharides such as amylose, amylopectin, glycogen, and cellulose have many free reactive hydroxyl groups [45]. There are some polysaccharides that have hydroxyl and carboxylic acid groups, which can be readily altered/modified [46].

### 3.2. Polysaccharide-Based Drug Delivery Systems Classification

Polysaccharides can be categorized into two groups: polyelectrolytes and nonpolyelectrolytes. Polyelectrolytes can be further divided into three subgroups based on their intrinsic charge: cationic (chitosan), anionic (alginate, heparin, pectin, hyaluronic acid), and neutral (pullulan, dextran) [47].

There are various classifications of polysaccharides based on the Source of Origin, including [48]:

− Plant/algal
− Starch, which includes amylose, amylopectin, cellulose, agar, alginate, and carrageenan.
− Pectin and konjac, as well as guar gum.
− Animal-based substances, such as chitin/chitosan, and hyaluronic acid.
− Bacterial substances, such as xanthan, dextran, levan, and curdlan.
− Fungal substances, including pullulan and yeast glucans.

Next, in Table 2, an overview of main polysaccharide-based drug delivery systems discussed in this review is presented.

Next, some recent, important studies that can be found in the specialized literature, related to the subject of main polysaccharide-based drug delivery systems are discussed. For each formulation case, the stage of study development and the administration route of a specific polysaccharide-based coating are mentioned.

### 3.3. Starch-Based Coatings as Drug Delivery Systems

Starch is made up of two different polymers: amylose and amylopectin. Amylose is a linear polymer, while amylopectin has a branching structure. The proportion of amylose to amylopectin in starch can differ depending on where it comes from. Advantages and disadvantages of starch designed as coatings are represented in Table 3.

Briefly, starch is a polymer made from renewable resources, which is nontoxic and biodegradable. However, its use in medicine is limited due to some drawbacks. By carboxymethylating starch, its water solubility is increased, and it becomes pH-sensitive, making it suitable for delivering pharmaceuticals in the desired form. For example, a starch included in a gel formulation was used as a thin-film coating for cardiovascular stent applications and tested in vitro. The article reports favorable chemical and crystalline changes, as shown by FTIR, HNMR, XRD, and TEM. A SEM analysis yielded a thin-film thickness of 1.4 ± 0.2 μm and electrosprayed droplet size of 172 ± 45 nm. Biocompatibility studies of HUVEC and L929 cells revealed appropriate results. Nanocomposite-coated surfaces were found to be compatible with blood, indicating their potential use in drug delivery in medical applications [71]. Carboxymethyl starch (CMS) has an anionic character and glycoside bond in its structure, which makes the drug-release behavior sensitive to enzymes, salt, and pH in CMS-based systems. To fabricate an effective drug carrier for a specific drug and organ of the body (such as the colon), it is important to consider the effects of these parameters on the carrier structure and release profile. Additionally, combining CMS-based systems with other stimuli-responsive polymers, inorganic materials, and photoluminescent materials can improve the efficiency of drug delivery systems [37]. Starch is a type of complex carbohydrate made up of glucopyranose residues in a specific linkage that breaks down into D-glucose when hydrolyzed. When glycidyl methacrylate is present, soluble starch in saturated aqueous solutions can react to form methacrylate-grafted starch. These grafted starch solutions can then be polymerized to develop hydrogels, either with or without the addition of an unsaturated acid. In Micale et al.’s study, polyphenol-loaded hydrogels were analyzed for local injections and oral administration with an emphasis on therapeutic benefits and innovative design and characterization studies [72]. Recent research has shown that the starch solutions and hydrogels produced through grafting can be broken down by the α-amylase enzyme. These acidic hydrogels have potential applications as protective coatings that can be enzymatically degraded in self-regulating drug delivery systems [73] and it has been discovered that the pH level of an acidic starch hydrogel is necessary for the efficient functioning of this self-regulated drug delivery system. This suggests that it is possible to fabricate a hydrogel that is suitably acidic for this purpose. Studies on hydrogel dressing showed that it can protect injured skin and speed up the healing process by keeping it moist and biocompatible with L929 fibroblast cells [73]. The research community has expressed concern about the use of native starch in certain controlled drug delivery systems due to the potential for drugs to be released too quickly from such systems. The use of antimicrobial agents in starch-based coatings can be achieved through various methods such as microencapsulation, electrostatic spinning, and direct incorporation. Characterization and cellular studies have shown that these antibacterial starch-based materials can be utilized not just as food antibacterial agents but also extensively in the packaging industry in the form of films [74]. The reason for this is because starch undergoes extensive swelling and quick enzymatic breakdown in living organisms.

Generally, starch derivatives are less susceptible to enzymatic breakdown compared to natural starch. Starch–chitosan complexes have promising potential as carriers for pharmaceuticals. Nanoparticles composed of oxidized starch and chitosan have been assessed for their ability to carry anti-infectives and nucleic acids. Transfection studies have shown approximately 5% of A549 cells with reporter gene expression [75]. Over the past decade, hydrophilic and hydrophobic starch derivatives have been utilized to produce drug delivery systems such as microparticles, nanoparticles, nanocrystals, hydrogels, and scaffolds. Modifying the surface of starch nanoparticles through cross-linking and esterification reactions represents a promising method for producing innovative, intelligent solid dosage forms. Oral drug delivery systems are currently exploring the use of starch derivatives. For example, characterization and cellular studies of Ref. [76] have shown that crosslinked starch, starch-g-poly(acrylic acid) copolymer, and starch/(ethylene-vinyl alcohol copolymer) can be effective in reducing inflammation and delivering peptide drugs. Meanwhile, other researchers have explored ways to mitigate the impact of acetylation on the swelling and enzymatic breakdown of corn starch. They have also analyzed the release rate of drugs from acetylated starch-coated tablets and their ability to target the colon. Thus, Ling Chen et al. [49] explored the impact of acetylation on corn starch. They looked at how acetylation affects swelling, enzymatic degradation, and drug release from starch-based polymer-coated tablets. The researchers prepared corn starch acetates (SA) with varying levels of acetyl esterification (1.31 to 2.40). Bovine serum albumin was used as a model drug. In vitro drug release assays were conducted with simulated gastric fluid, simulated intestinal fluid, and simulated colonic fluid. This study found that acetylation increased the resistant starch content and swelling capacity of corn starch. The whole process delayed its enzymatic degradation. Since tablets coated with SA were able to deliver the drug into the colon, it was suggested that SA could be a viable drug delivery vehicle for colon-targeted biomacromolecule drugs [49].

It has been discovered that starch capsules produced through the injection molding process serve as a valuable alternative delivery mechanism for active agents that are orally dispensed [77,78]. Skin diseases and damaged skin require novel drug delivery systems for effective topical treatment. Results on drug administration, in vitro, and in vivo experiments highlight the advantages and effective therapeutic capabilities of the use of nanoparticles for burns, wound healing, scar prevention dermal infections, and atopic dermatitis [77]. Starch capsules synthesized by process of injection molding, have proved to be a very useful alternative delivery system for orally administered compounds and to target drug to specific sites in the human gastrointestinal tract. Scintigraphy results confirmed the in vivo behavior of starch capsules compared with traditional hard gelatin capsule [78].

The pharmaceutical industry finds biocompatible and bio-based materials to be highly desirable resources. One such material is Poly(glycerol adipate) (PGA), a polymer that is both biocompatible and biodegradable. PGA can be utilized to fabricate self-assembled nanoparticles (NPs) that can effectively encapsulate drugs, showing promising potential for drug delivery. Additionally, starch is a versatile, inexpensive, and widely available polysaccharide that can function as bio-scaffold for other molecules, providing them with new and desirable properties. In the investigation of [79], the researchers found a successful method for controlled release of hydrophobic drugs, using a combination of PGA NPs and starch films as a biopolymeric matrix carrier. The size of drug-loaded PGA NPs was determined using dynamic light scattering (DLS), while UV–Vis spectroscopy was used to assess the enhancement of the drug water solubility. Biological assays were conducted on cancer cell lines and bacterial strains to confirm the effectiveness of drug-loaded PGA-NPs. To monitor the NP release profile during starch/PGA nanocomposite film digestion, dye-conjugated PGA was used, and the digestion models that mimic physiological conditions were evaluated. In vitro biological assays obtained in the case of a biodegradable carrier system for oral drug delivery confirm that drug-loaded PGA NPs maintained the effective activity of the therapeutic agents [79]. Maryam Moghadam et al. [80] developed a new light curable starch-based hydrogel drug delivery system to improve the release rate of quercetin as a poorly water-soluble drug. To increase the release rate of Quercetin, they developed a starch-based hydrogel that was light-curable and eco-friendly. They synthesized a modified starch-based hydrogel with high porosity, which was cross-loaded into the hydrogel structure before cross-linking, resulting in highly efficient drug absorption. The hydrogel structure was improved by adding hydrophilic glycol compounds, which increased the cross-release rate to 56.62% after 8 h, almost double compared to the previously reported study. However, further in vivo studies are needed to evaluate the bioavailability of the synthesized compound, which have potential for tissue engineering, cell encapsulation, wound healing, cardiology, and drug delivery applications [80].

A new method for improving drug delivery to the colon has been developed using a porous scaffold based on starch-based carrier coated with chitosan–phytic acid [81]. The carrier was designed for oral administration and was tested using hydrophobic paclitaxel as a model drug. By adjusting the size, shape, and adsorption power of the porous starch, drug loading and entrapment efficiency were significantly improved. The addition of chitosan–phytic acid provided a secondary layer of protection, which was necessary due to the low dissolution rate of porous starch during simulated digestion in the stomach and small intestine. However, the release curve showed that a high percentage of the drug was released in the colon. The study also confirmed the noncovalent interactions between starch and paclitaxel, indicating that the binding between the two is due to hydrogen bonding and the hydrophobic effect of CH-π [81].

Polymeric films containing pomegranate peel extract based on PVA/starch/PAA blends for use as wound dressing were evaluated for physicochemical properties and tested in vitro. It has been suggested that films can be produced using a combination of poly(vinyl alcohol), starch, and poly(acrylic acid)—polymers commonly found in pharmaceutical excipients—along with pomegranate peel extract (PPE). PPE contains bioactive compounds with antimicrobial and healing properties, making it an ideal component for a bioactive wound dressing [82]. An in vitro method was used to examine the minimum inhibitory concentration (MIC) of PPE. The best concentration of PPE for film fabrication was identified through antimicrobial susceptibility tests using the diffusion disc method. Films were prepared through the solvent cast method with two concentrations of PPE: 1.25% and 2.5% *w*/*v*. The lower concentration of PPE showed statistically insignificant differences in antimicrobial activity against *Staphylococcus aureus* (*S. aureus*) and *Staphylococcus epidermidis* (*S. epidermitis*) compared to the higher concentration. Therefore, the films made with the lower percentage of PPE (1.25% *w*/*v*) were chosen for further studies. The miscibility and stability of extract in the film forms were examined through thermal analysis, while parameters determining the barrier properties of the films were studied through complementary techniques. In vitro biological tests were conducted to assess safety and activity. Results showed that films with a higher amount of starch (15% *v*/*v*) were smooth, transparent, and domain-free, with no phase separation. The film also exhibited barrier properties suitable for use as a coating. These films were nonhemolytic and biocompatible when subjected to the in vitro hemolytic activity assay, with no observed toxicity of the extract at the tested concentrations. Wound healing in vitro tests revealed that films containing 1.25% PPE efficiently reduced scratch open areas and produced almost complete closure of the scratches within 48 h without cytotoxicity [82].

Another study examines the antidiabetic impact of fucoxanthin, encapsulated in porous starch (PS), on mice with type 2 diabetes induced by streptozotocin and nicotinamide [83]. It was conducted to examine the effects of fucoxanthin, extracted and purified from *Sargassum angustifolium*, when encapsulated in porous starch (PS) on diabetic mice. The mice were given either free fucoxanthin (400 mg/kg) or fucoxanthin-loaded PS, along with metformin (50 mg/kg), daily for three weeks. The results showed that both forms of fucoxanthin significantly prevented weight gain in the treated groups (*p* < 0.05) and were able to lower fasting blood glucose and increase plasma insulin levels similar to metformin (*p* < 0.05). Total cholesterol, triglycerides, and low-density lipoprotein were also lower in the treated groups, indicating the antiobesity effect of fucoxanthin through the regulation of lipid profile parameters. Additionally, the oral administration of metformin and fucoxanthin caused regeneration of pancreatic beta cells, as evidenced by histopathological evaluation of pancreatic tissue in diabetic mice. The study confirms that oral administration of fucoxanthin in STZ-induced diabetic mice has an appreciable antidiabetic effect and can reduce lipid profile parameters, even leading to pancreatic beta cell regeneration. These findings suggest that fucoxanthin, a bioactive compound found in algae dye, can be used in functional foods for natural diabetes therapy [83].

### 3.4. Chitosan-Based Coatings as Drug Delivery Systems

Chitosan is a natural cationic polymer that is biocompatible and biodegradable. It can be modified through different reactions to produce various derivatives with different structures, properties, and functions. Chitosan and its derivatives have been extensively researched and developed for transdermal drug delivery, showing unique advantages and disadvantages, as shown in Table 4.

A technique called dip coating was used to fabricate layers of chitosan and polycaprolactone (PCL) with microspheres containing vancomycin or daptomycin. This allowed for controlled delivery of antibiotics to fight infections associated with implants. To improve adhesion to metal substrates, the film surface was mechanically abraded. Studies have shown that the release of drugs depends on the type of drug, pH of solution, and whether the drug is encapsulated in PMMA microspheres or directly incorporated into films. For example, free-standing films of daptomycin showed 90% release after 1 day at pH 7.4 and 4 days at pH 5.5, while films with microspheres encapsulated in daptomycin achieved 90% release after 2 h at pH 5.5 and 2 days at pH 7.4. Similar results were found for vancomycin-encapsulated and free-standing films. Additionally, daptomycin-loaded films showed activity against susceptible and clinically isolated *S. aureus* strains when assessed by agar diffusion assays [51].

Visan et al. [85] developed for orthopedic application an effective way to fight infectious agents with their Antimicrobial Platform for Extended Release of Tetracycline. They used laser coating to apply an inorganic/organic composite mixture of amorphous calcium phosphate–chitosan–tetracycline onto surfaces. The functionalized surfaces have been shown to remain active for several days, making them a long-lasting solution. Mass loss and UV–VIS studies have demonstrated the sustained release of the drug (tetracycline) in simulated liquids. The drug release profile of the composite coatings has two stages: an initial sudden release followed by a slower development that is active for the next 72 h and probably longer. The optimized coatings strongly inhibit the growth of *Enterococcus faecalis* and *Escherichia coli* (*E. coli*) bacteria while maintaining an excellent biocompatibility and normal development of bone-like maintain MG63 cells. The proposed composition [85] of coatings has the potential to develop a new generation of antimicrobial coatings for medical implants and the prevention of nosocomial and large-scale contamination.

Mohsen M. Mady et al. [86] investigated the use of chitosan to implement a protective capsule for new drug formulations. Their study analyzed chitosan-coated liposomes for topical release in pharmacological applications using drug release rate, transmission electron microscopy, zeta potential, and turbidity measurements at 400 nm to optimize their characteristics. The results showed that chitosan increased the stability of liposomes during drug release. Coating liposomes with chitosan slightly increased their size by adding a layer of approximately 92 ± 27.1 nm. As the concentration of chitosan increased from 0.1% to 0.3% (*w*/*v*), the liposomal zeta potential became increasingly positive before stabilizing at a relatively constant value. By optimizing the properties of both liposomes and chitosan, specific, prolonged, and controlled release of liposomes can be produced [86].

Ophthalmic drug delivery systems using chitosan-based nanocoatings for intraocular implants, either alone or in combination with standard treatments, may be an effective way to prevent or treat acanthamoeba keratitis or endophthalmitis. However, further research is needed to determine the safety and efficacy of this approach. Specifically, randomized controlled trials on healthy eyes with soft or hard contact lenses or orthokeratology lenses for refractive error correction could help to assess the prophylactic benefits of this innovative drug delivery system. Other chitosan-based nanocoating ophthalmic drug delivery systems also warrant further investigation [87].

A recent study by A. Taherian and colleagues reported the use of a new extract made from black pomegranate peel and loaded with chitosan-coated magnetic nanoparticles. Core–shell nanoparticles were synthesized and used as a drug carrier for treating breast cancer cells. The researchers found that the nanoparticles containing the drug were highly effective in eradicating cancer cells, surpassing the efficacy of free drugs. Furthermore, both the drug-free and drug-loaded nanoparticles showed no toxicity to normal cells [88]. A different research project involved novel coatings containing cefazolin on titanium. These coatings were made using a drug carrier called chitosan, which is physically cross-linked and has properties that are safe for cells and allow for sustained drug release. Two types of coatings were made using this drug carrier: cefazolin/chitosan (P-Z@C) and cefazolin/chitosan crosslinked with calcium phosphate (P-Z@C/CP). The coatings were applied to titanium using pulsed direct current (DC) power. The characteristics of the coatings, including their microstructures, drug loadings, drug release rates, antibacterial properties, and effects on cells, were analyzed. Transmission electron microscopy images revealed that calcium phosphate granules were dispersed in a chitosan matrix in the P-Z@C/ CP coating. The granules had lower drug concentration than the surrounding chitosan. The (P-Z@C) coating had thrice the drug content (259.6 μg/cm²) than the P-Z@C/ CP coating (95.4 μg/cm²). Despite this, the drug release rate of the P-Z@C coating (75.0%) was slower than that of the P-Z@C/ CP coating (85.9%) after 30 days. The high sustainable drug-release ability of the P-Z@C coating was attributed to its low swelling ratio. Furthermore, there was no significant difference in cell numbers between the P-Z@C/ CP coating and titanium. However, the P-Z@C coating had fewer cell numbers than titanium. All the drug-containing coatings exhibited good antibacterial activity against *S. aureus* after 24 h [89].

Silver nanoparticles (AgNPs) in green color were produced using the metabolite of *Streptomyces malachiticus* and sunlight. These nanoparticles were coated with a mixture of curcumin and chitosan to produce a drug delivery tool for curcumin that would target the liver fibrosis mouse model induced by carbon tetrachloride (CCl4). Inducing fibrosis increased the expression of genes such as COL1A1, α-SMA, PDGFRB, and TIMP1, as well as affected hepatic enzymes, histopathological findings, and collagen deposition, which were all determined using Mason’s trichrome staining. Treatment with naked AgNPs had a tendency to increase the inflammatory effects, while coating them with chitosan did not prevent the fibrogenic effects of CCl_4_, as was observed with curcumin treatment. However, using curcumin/chitosan-coated AgNPs resulted in the reversal of liver fibrosis. Curcumin was found to be an effective antihepatic fibrosis drug in this nanoform, maintaining hepatic architecture and function during fibrosis development. Its effectiveness can be attributed to its inhibitory role, such as direct binding to fibrosis-mediating proteins such as PDGFRB, TIMP-1, TLR-9, and TGF-β [90].

Juliana Moraes Souza Arajo et al. reported the use of an edible chitosan coating made from cassava starch, enriched with *Lippia sidoides Cham.* essential oil (EO) and pomegranate peel extract. This coating was used to preserve Italian tomatoes (*Lycopersicon esculentum Mill*.) stored at room temperature [91]. The application of coatings on tomatoes showed to be effective in reducing weight loss and TSS levels, as well as delaying fruit ripening. Formulation 8, which included 10 g L^−1^ of CS, 10 g L^−1^ of CH, 10 mL L^−1^ of EO, and 20 mL L^−1^ of PPE, exhibited the best results with the lowest weight loss and reduced TSS content. These coatings were able to maintain the quality of Italian tomatoes during 12 days of storage at 25 °C, which is crucial for their perishability. The physicochemical characterization and microbiological quality results proved that the edible coatings are effective in preserving food by acting as a barrier against gases and water vapor, enhancing food appearance and preventing microbial contamination. However, further studies are necessary to evaluate the sensory parameters of coated tomatoes and the safety of *Lippia sidoides* EO and PPE for human consumption.

### 3.5. Dextran-Based Coatings as Drug Delivery Systems

Dextran is a natural polysaccharide that possesses outstanding properties for fulfilling essential nanomaterial needs in pharmaceutical applications. These properties include biodegradability, biocompatibility, and nontoxicity (Table 5).

Various routes exist for delivering therapeutic agents, including nasal, ocular, oral, parenteral, pulmonary, transdermal, and vaginal or anal. Drug delivery systems can also be designed based on the type of drug carrier materials, such as dendrimers, liposomes, hydrogels, micelles, quantum dots, nanomaterials, and mesoporous or polymeric systems. Additionally, chemotherapeutic drugs can be entrapped in other formulations such as a thermosensitive star-like copolymer. For instance, a water-soluble copolymer called dextran-graft-poly-N-iso-propylacrylamide was used to deliver toxic doxorubicin to cancer cells and showed higher toxicity compared to the free drug form, requiring a lower concentration to obtain a therapeutic effect. This formulation is a promising doxorubicin delivery platform [19]. Chemical modifications have resulted in the formulation of several derivatives of dextran, as it contains numerous reactive hydroxyl groups on its backbone. New delivery systems based on dextran, such as micelles, miniemulsions, magnetic nanoparticles, hydrogels, and spray-dried particles, are being developed. Their physicochemical properties, mechanisms of release, and therapeutic effects on animals have been extensively studied, making them highly applicable in biomedicine, particularly in cancer treatment. However, clinical studies have revealed unexpected side effects of dextran, such as thrombocytopenia and hepatotoxicity [19].

Many dextran derivatives are made using organic solvents and potentially harmful chemicals, which raises safety concerns. It is crucial to find environmentally-friendly manufacturing methods and learn more about how dextran-based nanoparticles behave in living organisms. More in vivo studies are needed to understand how these nanoparticles change in structure and metabolism under physiological conditions. This information will help to design better dextran-based delivery systems. Additionally, when testing drug-loaded dextran-based nanoparticles, intravenous injection is the preferred method of administration over oral administration [19]. It is widely acknowledged that the oral route is the most convenient method for drug delivery. Therefore, it is important to focus on developing dextran-based nanoparticles as effective oral delivery systems. These nanoparticles should demonstrate good stability, mucus permeability, and transepithelial transportability [19].

In the study conducted by Cristina Chircov et al. [93], the authors designed microscaffold matrices based on poly(lactic acid) (PLA) and graphene oxide (GO) via electrospinning, with quercetin (Q) loaded as a model drug for wound dressing applications. The PLA/GO/Q scaffolds showed uniform surface morphologies and fast release of Q under electrical stimulation. The scaffolds also demonstrated antimicrobial properties and biocompatibility with fibroblast cells, making them a promising platform for personalized drug delivery. New iron oxide scaffold nanocarriers were produced by adjusting the synthesis parameters (such as pressure and reaction time), using a microwave-assisted hydrothermal process. To make these carriers more stable and efficient in carrying curcumin, they coated them with a layer of dextran. These nanosystems, which come in different concentrations, can be used to coat implantable devices such as bone grafts, catheters, meshes, and wound dressings. This could help more patients who are at risk of infection. Haemanthamine (HAE) is a potential anticancer agent, but its therapeutic use is limited by its chemical instability and poor water solubility. To overcome these challenges, Nguyen et al. developed new amphiphilic electrospun nanofibers loaded with HAE, phosphatidylcholine (PC), and polyvinylpyrrolidone (PVP) for stabilizing liposomes of the active agent. The nanofibers were made using solvent-based electrospinning. The HAE-loaded fibers had a nanoscale size ranging from 197 to 534 nm, and liposomes with diameters between 63 and 401 nm were spontaneously formed when the nanofibers were exposed to water. HAE dispersed inside liposomes showed a trimodal dissolution behavior. Their results emphasize that amphiphilic nanofibers could be an alternative approach to formulating a liposomal drug delivery system and stabilizing the liposomes of the present alkaloid [94].

A new antibacterial coating was fabricated using carboxymethyl dextran (CMD) and microarcoxidized titanium (MAO-Ti) [56]. The coating was designed to release minocycline (MC) using self-assembled nanocells made of CMD and octadecylamine (ODA). The MC-loaded nanocells were crosslinked to improve their stability. The coating was successfully incorporated into the pores of MAO-Ti, making it more hydrophilic without affecting its roughness. The coating released MC over 360 h at a rate of 86.6%. The tests showed that the coating was effective against the bacteria *S. aureus* and was biocompatible with human skin fibroblasts. The coating reduced the number of *S. aureus* and improved the viability, adhesion, and morphology of the human skin fibroblasts compared to smooth titanium films/sheets. The MC-loaded CMD-based nanocells coated on a MAO-Ti surface (MC@(ODA-CMD)CL-Ti) have great potential as coatings for percutaneous implants due to their sustained-release properties, excellent antibacterial properties, and biocompatibility [56].

In our previous work devoted to biomedical applications, we developed thin films of dextran–iron oxide by dispersing iron oxide nanoparticles (15% concentration) and dextran–iron oxide (10% concentration) in distilled water. These solutions were frozen in liquid nitrogen and used as targets during matrix-assisted pulsed laser evaporation (MAPLE), with a KrF* excimer laser source (λ = 248 nm, τ_FWHM_ ≅ 25 ns, ν = 10 Hz) [95]. This process deposited continuous thin films with the same crystallinity, chemical composition, and molecular structure as the starting materials. Microscopic images showed that HepG2 cells grown on the dextran and dextran–iron oxide thin films from MAPLE targets with a low iron oxide NP concentration of 1 wt% formed slightly larger multicellular aggregates than those on the thin films from the 5 wt% iron oxide–NPs MAPLE target. Aggregate size increased with incubation time in all cases. These results, confirmed by viability tests, suggest good biocompatibility of all synthesized composite thin films [95].

Cerium-doped hydroxyapatite (Ca_10−x_Ce_x_(PO_4_)_6_(OH)_2_) coated with dextran was grown on Si substrates using high-frequency magnetron sputtering technique. The deposition process was first described by Ciobanu et al. Two variants were made: 5CeHAp-D with x = 0.05 and 10CeHAp-D with x = 0.10 [96]. The coatings produced were analyzed using various techniques, such as scanning electron microscopy (SEM), energy dispersive X-ray spectroscopy (EDX), atomic force microscopy (AFM), metallographic microscopy (MM), Fourier transform infrared spectroscopy (FTIR), and glow discharge optical emission spectroscopy (GDOES). The collected data regarding the surface morphology, composition, and structure were studied and discussed. The surface morphology of CeHAp-D composite thin films was found to be smooth without any granular structures. The constituent elements of CeHAp-D target were successfully identified. The FTIR measurements revealed the presence of peaks related to the ν 1-, ν 3-, and ν 4-vibrational modes of (PO43-) groups from the hydroxyapatite (HAp) structure, as well as those specific to the dextran structure. Human cells retained their specific elongated morphology after 24 h of incubation, indicating that the behavior and proliferation ability of gingival fibroblasts were not affected by the presence of 5CeHAp-D and 10CeHAp-D composite coatings. The surfaces of 5CeHAp-D and 10CeHAp-D coatings for dentistry were found to be harmless to human gingival fibroblasts, indicating their good biocompatibility [96].

Based on the results of polyaldehyde density, nanoparticle size, and functionality, it appears that dextran-coated nanoparticles have potential for use as immunosensing platforms. In immunoassays, it was found that polyaldehyde–dextran nanocarriers exhibit greater sensitivity compared to polycarboxylated dextran carriers [97].

Elsa Daz-Montes and her team proved that using a dextran/chitosan blend to make films can be an effective bio-packaging option for maintaining the freshness and quality of mushrooms, thus extending their shelf life. They found that the lowest dextran concentration (0.5% *w*/*v*) had the best properties for tensile strength, elastic modulus, water vapor permeability, and delayed spoilage time of mushrooms [98].

A fascinating study was conducted on the fabrication of photo-crosslinkable dextran-based hydrogel films. These films were designed with a specific structure to serve as scaffolds for osteoblasts and endothelial cells to adhere and grow on. To reinforce the structure, nanoparticles were utilized with benzophenone units as multiple photo-crosslinking moieties. Gelatin particles were also incorporated into the matrix to allow for cell growth and partial degradation of the hydrogel. The hydrogel films were characterized in terms of swelling behavior and structural properties to optimize their cell growth properties. By further functionalizing the hydrogel layers with BMP-2, scaffolds were produced to mimic the natural tissue environment for studying the growth of endothelial and osteoblast cell cultures. Characterization studies of functional hydrogel composite films allow independent selection and optimization of each component in the hydrogel composite for a wide range of potential applications for targeted cell growth, as successfully shown in Ref. [99] with osteoblast–endothelial cell co-culture for bone tissue regeneration.

### 3.6. Hyaluronic Acid-Based Coatings as Drug Delivery Systems

**Figure 4:**
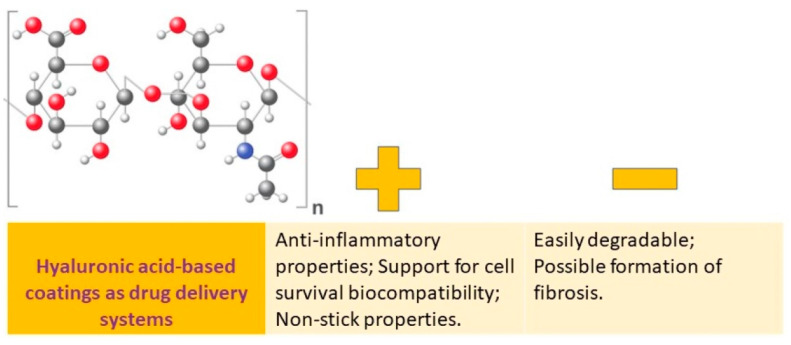
Hyaluronic acid-based coatings as drug delivery systems: advantages vs. disadvantages.

Hyaluronic acid (HA) is a significant component of the extracellular matrix (ECM) and is a large, nonsulfated glycosaminoglycan (Figure 4).

Multiple formulations of hyaluronic acid (HA) and its derivatives have been developed, including coatings, hydrogels, and nanoparticles. These formulations have numerous benefits due to the properties of HA, which include anticoagulant, anti-inflammatory, antiproliferative, immunomodulatory, targeted transport, sustained release, and cell compatibility. These formulations have been regularly utilized in areas such as tissue engineering, wound healing, and cancer treatment [19].

HA can be used for targeted drug delivery in cancer treatment, skin disorders, wound healing, and inflammatory arthritis. CD44 and RHAMM receptors help with receptor-mediated endocytosis of HA, making it more effective in delivering drugs to cancer cells. Drug delivery using HA is a novel approach that relies on the function of receptors such as CD44 and RHAMM to facilitate receptor-mediated endocytosis of HA within the body. This technique is especially beneficial in cancer treatment, as it has been demonstrated that HA delivery systems can enhance the cellular uptake and effectiveness of drugs targeted at tumors [100].

In Zhao et al.’s work, a highly lubricated coating was designed and applied to the tracheal tube. They developed a coating made from hyaluronic acid that is ultra-slippery, nonirritating, and anti-inflammatory. This coating was designed to reduce the risk of injury during intubation procedures [101]. On the surface-activated tubing, a layer of hydration was formed through the intermolecular interaction of hyaluronic acid and triblock copolymer (Pluronic F127) being codeposited. This coating has a high artificial saliva adsorption ratio and water retention rate, which makes the tube perfectly lubricated. When compared to the original tube, the coefficient of friction of the coated tube in artificial saliva was 77% lower. The effectiveness of the lubricated coating in endotracheal intubation was evaluated in vivo using a cynomolgus monkey model. The coating was found to be effective in alleviating the injuries caused by endotracheal intubation [101].

Hyaluronic acid-based hydrogel coatings on Ti6Al4V implants have excellent in vivo biocompatibility, promote cell proliferation, differentiation and mineralization, and provide sustained drug release. On the surface of the Ti6Al4V biomaterial, hydrogel coatings based on hyaluronic acid were produced using crosslinkers such as 1,4-butanediol diglycidyl ether (Ti-HABDDE) and divinyl sulfone (Ti-HADVS) [102]. Hydrogel coatings have been found to be highly compatible with living tissues, promoting the growth, differentiation, and mineralization of cells while also being able to sustain drug release. Additionally, they exhibit multifunctional antibacterial activity, repelling 51–55% of *S. aureus* and 27–40% of *E. coli* and killing 82–119% of *S. aureus* and 83–87% of *E. coli* as drug-loaded hydrogel coatings with bactericide release (R > 2) [102].

A recent study focused on developing free-standing and self-healing coatings through the layer-by-layer (LbL) self-assembly method. A self-healing coating made with hyaluronic acid and silk has been developed for tissue repair that also has antibacterial properties. Such coatings consist of beta-cyclodextrin-modified silk fibroin (SCD) and adamantane-modified hyaluronic acid (HAD) and act based on host–guest interactions. The coatings possess the ability to repair external mechanical damage and be removed from the substrate repeatedly. Furthermore, they display remarkable antibacterial properties and biocompatibility. Additionally, the proliferation and myelination of Schwann cells are improved. That study highlights the potential of multifunctional coatings in tissue engineering, particularly in nerve regeneration [103]. An in vitro study conducted in a controlled environment displayed the effectiveness of an antibacterial hydrogel coating on a titanium disk. The coating contained a 28% (*w*/*v*) concentration of polymer and 1–2% (*w*/*v*) of vancomycin or tobramycin or their compounds. This coating was able to release the active substances for up to 72 h in a time- and dose-dependent manner. The amount of active substance released was 100–1000 times higher than the minimum inhibitory concentration. Similar results were observed with other antibacterial compounds or their combinations, such as vancomycin, teicoplanin, rifampicin, daptomycin, tigecycline, cefazolin, gentamicin, tobramycin, amikacin, meropenem, and levofloxacin at concentrations ranging from 2–10% [58].

A hydrogel coating has been developed using hyaluronic acid and presented antibacterial properties for implantable biomaterials used in orthopedics and trauma. It is a patented hydrogel coating, which is commercially available and based on HA grafted to PLA, is called DAC^®^ or “Defensive Antibacterial Coating”. It is provided by Novagenit Srl in Mezzolombardo, Italy, and can be found at www.coatingdac.com or www.dac-coating.com. The kit comes with a prefilled syringe containing 300 mg of sterile DAC powder that is filled with a solution of 5 mL sterile water for injections during surgery. The desired antibiotic is mixed with the solution to obtain the antibiotic-loaded hydrogel with a DAC concentration of 6% (*w*/*v*) and an antibiotic concentration of 20 to 50 mg/mL, depending on the surgeon’s selection, in approximately 35 min. Surgeons can select from a list of previously tested antibiotics that are compatible with the hydrogel. After reconstitution, the hydrogel can be directly applied to the implant and inserted into the body, as usual. If necessary, the hydrogel can be left at ambient temperature for up to 4 h after reconstitution [104].

Microneedles made of Hyaluronic Acid (HA) can help to transport different types of molecules, including adenosine and bioactive proteins to catalyze collagen and elastin production [105]. Transdermal application of alendronate via self-dissolving microneedles in rats was emphasized in [106]. The results on pharmacological effects and absorption evaluation proved that can help in delivering alendronate for osteoporosis treatment [106], and insulin for diabetes management [107].

Hyaluronic acid coatings have been developed for local drug delivery via implants. The main goal of authors was to design implants with a hydrophilic lubricating coating that could also provide local drug delivery (LDD). To achieve this, they worked on developing a stable and well-adherent HA coating that would have consistent drug uptake and release properties on PA12 model implant surfaces. The research team aimed to tune the release properties using two different crosslinking methods, as these coatings would be useful for both spontaneous and sustained LDD. To attach the HA base layer, functional groups that could covalently bind were generated using plasma and wet-chemical methods to activate the PA12 surface. This ensured that subsequent HA layers deposited by manual dip coating would adhere well [108].

### 3.7. Arabinogalactan-Based Coatings as Drug Delivery Systems

Arabinogalactan is a complex molecule made up of a galactan backbone and side chains of galactose and arabinose. It is highly soluble in water, with a solubility rate of up to 70%. This compound is derived from the Larix tree and is available in a pure form that is 99.9% reproducible in both molecular weight and physicochemical properties. Arabinogalactan can be found in powder and aqueous solutions and has been tested in the pharmaceutical industry as a binder and drug delivery agent for the colon. Once it is enzymatically broken down, the porosity generated by arabinogalactan is ideal for controlled drug release. Due to its high water solubility, biocompatibility, and biodegradability, arabinogalactan shows potential as a drug carrier [109].

**Figure 5:**
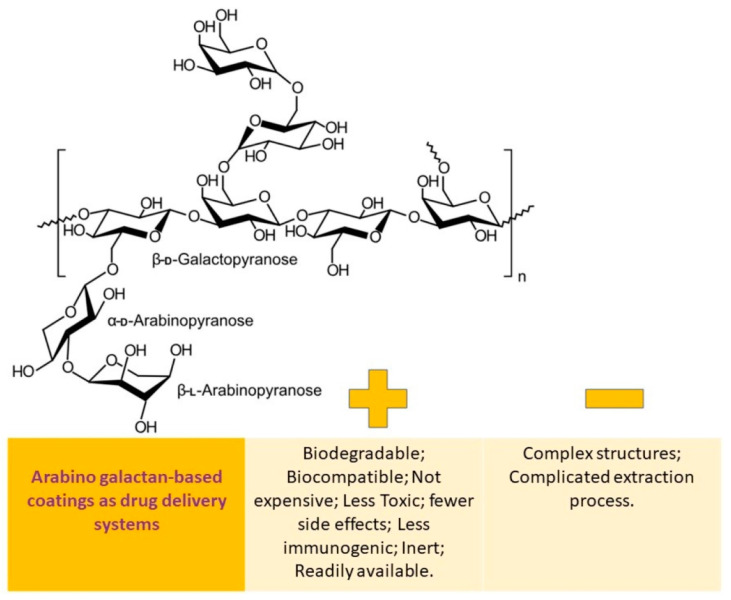
Arabinogalactan-based coatings as drug delivery systems: advantages vs. disadvantages.

The benefits and drawbacks of utilizing arabinogalactan-based coatings as a means of drug delivery are schematically depicted in Figure 5.

Researchers synthesized the cisplatin–arabinogalactan–aptamer (Cis-AG-Ap) conjugate using cis-dichlorodiamineplatinum, Siberian larch arabinogalactan, and aptamer AS-42 specific for heat shock proteins (HSP) 71 kDa (Hspa8) and HSP 90-beta (Hsp90ab1). They evaluated the antitumor effect of Cis-AG-Ap using ascites and metastatic Ehrlich tumor models, and also assessed its toxicity by conducting blood biochemistry tests on healthy mice. Their study demonstrated that Cis-AG-Ap had enhanced anticancer activity and specifically accumulated in tumor foci. Targeted administration allowed for a 15-fold reduction in the therapeutic dose of cisplatin, thereby reducing its toxicity. Cis-AG-Ap also successfully suppressed Ehrlich’s ascites carcinoma growth and the extent of tumor metastasis in vivo. The use of arabinogalactan and aptamers improved the bioavailability and efficiency of cisplatin, making this strategy very promising for targeted cancer therapy [62].

In the study of Avramoff et al., a once-daily controlled release formulation for diltiazem using arabinogalactan as a channeling agent was proposed. This formulation consists of two coated tablets in a capsule. The first tablet quickly releases, while the second one has a unique controlling membrane with arabinogalactan to achieve a delayed release profile for diltiazem. The in vitro characteristics of the formulation were determined by testing both the surface morphology of the coated tablet and impact of different polysaccharides on coating. Arabinogalactan was found to be the best channeling agent for controlling in vitro drug release. By adjusting the thickness of the outer shell and concentration of arabinogalactan, the drug can be released in a time-dependent manner. The formulation produced a desired delayed controlled release dissolution profile for over 24 h in buffer (pH 6.8). The surface morphology of the coating film showed channeling formation upon contact with the media [109].

Pinhassi et al. conducted a study that demonstrated the formation of a bio-macromolecular nanovehicle by linking folic acid (FA) and the cancer drug methotrexate (MTX) to arabinogalactan (AG). This nanovehicle can deliver a cytotoxic cargo specifically to cells that overexpress folate receptors (FR). Their study also showed a target-activated release mechanism by linking MTX with an endosomal cleavable peptide (GFLG). The FA-AGGFLG-MTX–drug conjugate had a 6.3-fold increase in cytotoxic activity against FR-overexpressing cells compared to their FR-deficient counterparts. This discovery presents a new polymer nanoconjugate for the targeted delivery of antitumor drugs into cancer cells. The bio-macromolecular drug carrier has the potential to selectively deliver and release chemotherapeutic agents for effective treatment of malignant cells, including multidrug resistant tumor cells. An important advantage of this polymer vehicle is that it can load multiple synergistically active antitumor agents in optimal stoichiometric ratios [110].

In addition, a study on drug conjugate of folic acid and arabinogalactan for targeted delivery and release of anticancer drugs to folate receptor-overexpressing cells proved that a vehicle of this nature can be customized to target a particular illness or individual, which is a crucial first step towards personalized healthcare [111].

Back in 2014, Kalpana N. Thakare and David B. Lebo patented a solid dosage form that contained arabinogalactan. This form allowed for a therapeutic agent to be delivered through a polymer matrix that contained arabinogalactan and a therapeutic agent that was evenly dispersed within it. The dosage form could be in the form of a microsphere, nanosphere, powder, tablet, film, or pellet that was encased within a capsule. The patent also included methods for producing this dosage form [112]. When drugs are targeted to the liver, they can minimize side effects by reducing distribution to nontarget organs and increase their effectiveness by concentrating them in the target cells. In a recent study, natural ligands such as arabinogalactan (AG), pullulan (PL), and lactobionic acid (LA) were chosen to target the asialoglycoprotein receptor (ASGPR-1) present on hepatocytes. The binding affinities of new ligands, including palmitoylated AG-(PAG), lauroylated AG-(LAG), palmitoylated PL-(PPL), lauroylated PL-(LPL), and lactobionic acid–adipic acid dihydrazide conjugate (LAD), were compared to AG, PL, and LA using in silico docking studies. These new ligands were successfully synthesized and characterized. They were incorporated into drug-loaded nanostructured lipid carriers (NLCs) for surface functionalization. The study examined HepG2 cellular internalization of hepatocyte-targeted NLCs using fluorescence microscopy and examined LAD-decorated drug-loaded NLCs that gave maximal cellular uptake using confocal microscopy. In vivo investigations evaluated the toxicity potential of LAD-decorated NLCs. Molecular docking results showed that LAD had the strongest binding affinity among all the ligands. Acute toxicity studies demonstrated the hemocompatibility and lack of organ toxicity for the ligand LAD. The study supports the proof of concept of the improved targeting efficacy of novel ASGPR targeting ligands. These ligands can be used to surface-modified nanocarriers for future targeted delivery in the treatment of various liver diseases [113].

Polysaccharide arabinogalactan was used to synthesize inclusion complexes of salicylic acid (SA) and acetylsalicylic acid (ASA). These complexes were studied for their physicochemical properties both in solid state and aqueous solutions, as well as their antiaggregation and ulcerogenic activity. Inclusion complexes of SA and ASA with the polysaccharide arabinogalactan (AG) from larch wood *Larix sibirica* and *Larix gmelinii* were synthesized using mechanochemical technology [114]. In their study, the authors conducted research on the characteristics of synthesized complexes in both solid and aqueous states. They also looked into the antiaggregation and ulcerogenic activity of these complexes. The nuclear magnetic resonance (NMR) relaxation technique was used to provide evidence of complex formation. In aqueous solutions, it was found that the molecules of SA and ASA rapidly exchanged between the complex with AG macromolecules and solution. The stability constant of aspirin complex was calculated. It was discovered that the mechanochemically synthesized complexes were more stable than the ones obtained by mixing solutions of components. ASA complexes showed a twofold increase in antiplatelet activity, which could lead to a decrease in the dose of antithrombotic drug and its ulcerogenic activity. These findings suggest that there is potential for developing new ASA-based preparations with increased activity and safety [114].

In a different research project, scientists studied the pharmacological and pharmacokinetic characteristics of ibuprofen (IBU), a nonsteroidal anti-inflammatory drug, combined with the natural polysaccharide arabinogalactan (AG) supramolecular complex. The aim of this combination was to enhance the bioavailability of ibuprofen and reduce its required dosage when administered orally. After being complexed with AG, ibuprofen effective analgesic and anti-inflammatory dose decreased by half due to the increase in ibuprofen concentration in rats’ plasma. The Cmax of IBU at doses of 20 and 40 mg/kg was found as 0.088 and 0.132 μg/mL, respectively, while the Cmax of IBU in the complex form was 0.103 and 0.160 μg/mL, respectively. Thus, the complexation of IBU with AG results in an increase in its bioavailability and reduction in the effective dose, which should decrease toxic side effects [115].

### 3.8. Pullulan-Based Coatings as Drug Delivery Systems

**Figure 6:**
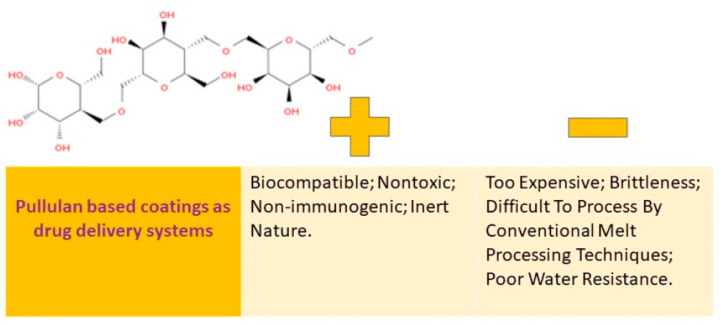
Pullulan-based coatings as drug delivery systems: advantages vs. disadvantages.

Many people are interested in pullulan because of its ability to stimulate the immune system, its ability to break down naturally, its compatibility with living tissue, and its water-loving properties. This substance is known as a biopolymer that can be altered through chemical processes, which is a promising way to broaden its uses. A visual representation of the pros and cons of using coatings made from pullulan can be found in Figure 6.

Pullulan is a type of homopolysaccharide that is composed of α-(1→4)–maltotriosyl (3-D-glucopyranosyl) units that are linked by α-(1→6) bonds. It is neutral in nature and is available as an amorphous mucilage substance that can be obtained from the aerobic fermentation broth of the *Aureobasidium pullulans* fungus. The molecular weight of this exopolysaccharide can vary from 4.5 × 10^4^ to 6 × 10^5^ Da depending on the yeast cultivation parameters [19].

Several derivatives can be synthesized from pullulan due to its flexible chain and the presence of hydroxyl functional groups that are prone to chemical modification. These derivatives include pullulan derivatives with cholesterol, pullulan acetate, carboxymethyl pullulan, pullulan succinylate, and pullulan amine. For example, in Ref. [116], cationic pullulan-based polymers for drug delivery with hydrophilic or amphiphilic properties were prepared and characterized. All the polymers were analyzed for their chemical composition, molar mass, thermal properties, critical aggregation concentration, and cytotoxicity. The graft copolymerization was confirmed by NMR and the degree of substitution for each copolymer was 0.13, 0.54, and 0.63, respectively. The results indicate that the physicochemical properties of PULL-DEAE and PULL-DEAE-g-PZLL could broaden the use of modified pullulan for pharmaceutical applications, such as drug delivery. In vitro cell tests showed no cytotoxicity even after prolonged exposure of the polymers to L929 MEF cells, which is a promising result for the future use of the copolymers as drug delivery systems. The development of cationic PULL-DEAE and PULL-DEAE-gPZLL by the methods described in this study is an important contribution to future research on the use of hydrophilic and hydrophobic pullulan derivatives for various controlled drug release devices, such as orodispersible films, solid particle suspensions for oral and topical administration, and particles in aqueous suspensions for intravenous administration [116].

By increasing its solubility in organic solvents or enriching it with new reactive functional groups, pullulan nonreducing agent, water solubility, formation of oxygen-impermeable films, hygroscopicity, biodegradability, and viscosity can be improved [116].

Cristescu and team recently announced the successful fabrication of thin films for medical implants using cinnamate–pullulan polysaccharide through matrix assisted pulsed laser evaporation (MAPLE) with potential pharmaceutical applications. The process was performed in a vacuum environment using a KrF* excimer laser source with a wavelength of 248 nm and repetition rate of 10 Hz, with each pulse lasting around 20 nanoseconds. They investigated the physical and chemical properties of thin films and emphasized how the surface morphology of the films was affected by the amount of laser energy used [117]. In a following study, the use of Raman spectroscopy confirmed that the thin films of cinnamate–pullulan medical surfaces, deposited through MAPLE, contain only the original materials and maintain their chemical structures without any impurities proving their potential use in targeted drug delivery systems and tissue engineering [118].

A group of researchers, led by Le, has developed new derivatives of polycationic pullulan that possess remarkable mucoadhesive properties and can release drugs for a long time to mucosal tissues. They activated the hydroxy groups of pullulan by using mesyl chloride and then linked them with low molecular weight polyamines. They evaluated two types of pullulan derivatives, namely, pullulan-tris(2-aminoethyl)amine (Pul-TAEA), and pullulan–polyethyleneimine (Pul-PEI), for their swelling behavior, mucoadhesive properties, and ability to control drug release. Pul-TAEA and Pul-PEI displayed excellent swelling properties at a pH of 6.8, exhibiting a weight gain of 240- and 370-fold, respectively. When compared to unmodified pullulan, Pul-TAEA and Pul-PEI showed a 5- and 13.3-fold increase in dynamic viscosity in mucus. Mucoadhesion tests of Pul-TAEA and Pul-PEI on the intestinal mucosa revealed a 6- and 37.8-fold increase in tensile strength, respectively, and a mucoadhesion time of 72- and 120-fold longer than unmodified pullulan. By using additional ionic interactions between the cationic groups on polyaminated pullulanes and anionic model drug, they achieved sustained drug release. Polyaminated pullulanes are a promising new type of mucoadhesive adjuvants that can be used in mucosal drug delivery [119]. Their study successfully synthesized cationic pullulan-based polymers with hydrophilic or amphiphilic properties through nucleophilic exchange and ring-opening polymerization. The first step involved pullulan partially modified with 2-chloro-N,N-diethylethylamine (DEAE) to obtain DEAE-modified pullulan (PULL-DEAE), which is both cationic and hydrophilic. The second step involved reacting the remaining hydroxyl groups of PULL-DEAE with N-carbobenzyloxy-1-lysine-N-carboxyanhydride (Lys(Z)-NCA) to form the grafted copolymer pullulan-DEAE-g-poly(Z-1-lysine) (PULL-DEAE-g-PZLL), which is a copolymer with cationic and amphiphilic properties. The copolymerization processes were carried out with different monomer concentrations (25, 40, and 55% by weight based on pullulan), resulting in three copolymers with hydrophobic segments of varying sizes. The polymers were characterized in terms of their chemical composition, molar mass, thermal properties, critical aggregation concentration, and cytotoxicity. NMR confirmed the graft copolymerization, and the degree of substitution for each copolymer ranged from 0.13 to 0.63. These findings suggest that the physicochemical properties of PULL-DEAE and PULL-DEAE-g-PZLL could broaden the potential applications of modified pullulan in fields such as drug delivery in the pharmaceutical industry [116].

Polymer films have many benefits when used to treat inflammatory skin conditions such as atopic dermatitis (AD). They can adhere to, protect, and hydrate the skin. Natural polysaccharides have proven to be a good choice for film development as they have low toxicity, biocompatibility, and biodegradability.

A recent study introduced a new approach for topical treatment of AD using a gellan gum/pullulan bilayer film with silibinin-loaded nanocapsules. The bilayer films were prepared using a two-step solvent casting process, with a layer of pullulan applied to a layer of gellan gum which incorporated silibinin nanocapsules. Microscopic analysis confirmed the bilayer formation. 

In vitro studies showed that pullulan provides bioadhesion to the films, and the nanocapsules significantly increased their occlusion factor. The nano-based film also demonstrated a slow silibinin release and high affinity to skin tissue, with high scavenger capacity and nonhemolytic properties.

In an in vivo study, vehicle film treatments reduced scratching behavior and ear edema in mice. However, the nano-based silibinin-containing film showed similar or more pronounced anti-inflammatory and antioxidative properties compared to silibinin solution and hydrocortisone, a classic treatment for AD. These results suggest that the gellan gum/pullulan bilayer film, combined with silibinin-loaded nanocapsules, can enhance the therapeutic effect on the AD model by protecting the skin from oxidative damage [64].

A new 3D film with porous properties has been launched for use in wound healing applications. This film is made of a chitosan/carboxymethyl pullulan polyelectrolyte complex (PEC) loaded with 45S5 Bioglass (CCMPBG) and was produced using the freeze-drying method. The chemical nature, microstructure, and surface morphologies of these films were analyzed using FTIR, XRD, EDS, and SEM. The CCMPBG films were found to have a rough surface morphology and well-interconnected micropores with an average size range of 10.1–74 μm. Compared to the chitosan/carboxymethyl pullulan (CCMP) control films, the CCMPBG films exhibited improved mechanical strength and controlled swelling and biodegradation behavior. This is due to the interaction between the polymer matrix and 45S5 Bioglass (BG). Additionally, the synergistic effects of chitosan, carboxymethyl pullulan (CMP), and BG resulted in improved biocompatibility, antimicrobial activity, and wound closure ability. Overall, these findings suggest that CCMPBG sheets may be an effective dressing material for wound therapy [120].

Films that aid in healing injuries and contain plant extracts have been shown to be effective in promoting skin regeneration. In this study, pullulan films were developed with an extract from the *Cyclospermum leptophyllum* plant to be used as healing dressings. The films were evaluated for their physical and chemical properties, as well as their potential antioxidant activity. The films were visually pleasing and flexible but showed some heterogeneity due to component precipitation. They were highly soluble and showed swelling, which could allow for better adhesion to wounds. Despite their rapid dissolution in water, the films could form a hydrogel and have controlled water vapor permeability, allowing for gas exchange. These biofilms have the potential for healing and future tests could demonstrate their antimicrobial and anti-inflammatory activities [121].

In the study by Popescu and colleagues’, orthotopic implantation of scaffolds made from alginate–pullulan–bioactive glass–ceramic with varying amounts of copper oxide was performed on experimental rat models to evaluate their efficacy in healing induced bone defects. Magnetic resonance and imaging scans were used in conjunction with histological evaluation to observe progressive bone healing within 5 weeks. As the regenerative process continued, new bone tissue was formed, enhancing the growth of irregular bone spicules around the scaffolds. Implantation of the composite with 1.5 mol % CuO (in glass–ceramic matrix) content resulted in a significantly higher amount of new bone formation (37%) in the defect. However, the bone regeneration obtained by scaffold with 0.5 mol % CuO implanted is comparable with the alginate–pullulan-β-tricalcium phosphate/hydroxyapatite composite implant. They found that all the compositions involved led to the formation of new bone between 29.75 and 37.15%. The involvement of alginate–pullulan composite materials in the regeneration process indicates their potential for use in tissue engineering [122].

The aim of this research was to investigate how adding pullulan to Eudragit NM-L55 blend film affects the properties of the resulting film, with a view to future drug release studies. Films were made using different ratios of simple polymers and blends, through an aqueous casting process. The resulting Eudragit NM-L55 and pullulan blend films were analyzed using infrared, mechanical, thermogravimetric, water vapor transmission rate, and swelling index studies. The studies showed that there were no intermolecular interactions between Eudragit NM-L55 and pullulan. However, increasing the proportion of pullulan up to 30% in the Eudragit NM-L55 blend film produced good quality films with enhanced tensile strength, increased modulus of elasticity, and decreased elongation at break. The thermal stability of the blend foils decreased up to approximately 400 °C with an increasing proportion of pullulan in the blend. Due to the high hydrophilicity of pullulan, water vapor permeability also increased with an increasing proportion of pullulan in the mixed films. Additionally, the addition of pullulan affected the swelling behavior of the blend films at pH 1.2 and 6.8. The higher ratio of pullulan in the mixed foils resulted in faster erosion of the foils. These findings highlight the significant impact of the polysaccharide pullulan on Eudragit NM-L55 blend film. It was discovered that a proportion of up to 20% pullulan in the synthetic mixed film is suitable for being applied as a coating material. Thus, adjusting the proportion of pullulan in the Eudragit NM-L55 blend film can result in suitable film properties [123].

### 3.9. Pectin-Based Coatings as Drug Delivery Systems

Pectin is a type of polysaccharide that exists in the cell walls of plants and aids in their growth [124]. This is a type of carbohydrate made up of polymers, which are mainly sourced from nature. It functions as a structural element in the cell walls of plants [125]. Pectin is a polysaccharide that is biocompatible and has different forms depending on its source or extraction method. It also possesses biological activity [125]. This is a substance called poly-14-galacturonic acid, which contains carboxylic acid residues that have been methylated to different degrees [125]. The degree of esterification of galacturonic acid is the most important factor that impacts the solubility and gelation properties of pectin [125]. Pectin can be used for various purposes due to its affordability, biodegradability, water solubility, and nontoxic nature [125].

**Figure 7:**
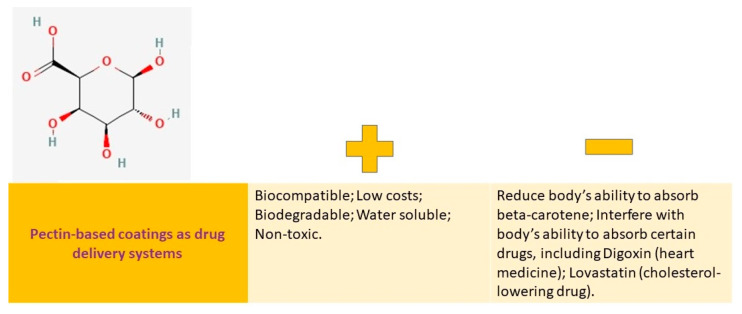
Pectin-based coatings as drug delivery systems: advantages vs. disadvantages.

Figure 7 provides a detailed explanation of the advantages and disadvantages of these options.

In recent years, a lot of attention has been given to biopolymer-based nanocomposites, specifically pectin nanocomposites, due to their exceptional thermal, mechanical, and biodegradable properties [125]. Pectin is a versatile substance that is utilized in various industries, including pharmaceuticals, cosmetics, food production, and biological research. Its popularity is due to its biocompatibility, biodegradability, and nontoxic properties [125].

In addition, pectin-based bio-nanocomposites have various applications in tissue engineering [125], wound [126], drug delivery [127], and cancer targeting [128]. Kaolin is frequently combined with it for drug delivery to the colon in oral formulations [125].

Several studies and reviews have demonstrated the versatility of pectin and its modified nanocomposites (NCs) for various applications. However, limited reports exist regarding their pharmaceutical and drug-delivery applications.

Polyelectrolyte complex nanoparticles (PEC NPs) were prepared by electrostatically interacting positively charged heat-denatured lactoferrin (LF) particles with negatively charged pectin. The obtained PEC NPs were utilized as curcumin carriers by encapsulating hydrophobic curcumin into LF/pectin PEC NPs, which showed high encapsulation efficiency and loading content. The in vitro controlled release and prominent antioxidant activities of curcumin from LF/pectin PEC NPs were observed. This method provides a facile and fast way to synthesize nanoscale food-grade delivery systems for improved water solubility, controlled release, and antioxidant activity of hydrophobic curcumin.

By incorporating 5 or 10% (*w*/*w*) pectin–chitosan and increasing the coating weight by 15 or 20% (*w*/*w*), the initial burst drug release was suppressed, and a delay phase of drug release was induced. In terms of drug burst in the colonic medium, formulations with a 20% (*w*/*w*) coating weight increase and 15% or 20% (*w*/*w*) pectin–chitosan exhibited superior performance compared to other formulations [129].

Buccal films are a type of drug dosage that can be easily applied and remain stable against microbiological contamination. They are effective in treating diseases of the oral mucosa, particularly those caused by infectious agents. Multilayer films, which consist of alternating layers of positively and negatively charged polymers, are an area of interest that has not been fully explored. In this study, the researchers aimed to develop antifungal multilayer systems using cationic chitosan and anionic pectin as potential platforms for the controlled release of clotrimazole. They characterized the systems pharmaceutically in terms of their release kinetics under different pH conditions, physical and mechanical properties, and mucoadhesive properties using an animal model of the buccal mucosa. They also evaluated their antifungal activity against selected *Candida strains* and their potential cytotoxicity to human gingival fibroblasts. Fourier transform infrared spectroscopy was used to investigate interactions between different polyions. The distribution of clotrimazole in the film layers had a significant impact on their in vitro dissolution profile. The designed films were found to be responsive to pH changes, with strong antifungal activity and a satisfactory safety profile. These findings suggest that these films could be useful in treating resistant cases of oral candidiasis, particularly when combined with chitosan to improve antifungal efficacy [68].

A study using different types of pectin as a film base for dosage forms (FDs) to assess drug dissolution in limited biological media was conducted. The authors analyzed the relationship between the decay profiles of FDs and the rates of drug release from them. They chose two pectins, A-PT and C-PT, as the film base, and used 24% of the base solution to prepare FDs with the right viscosity for casting. The FDs gradually degraded in the test medium, and the degradation of film matrix affected the dissolution rate of the drug in the FD. Their results suggest that FDs prepared using pectins are beneficial due to their high solubility in a limited amount of medium. Moreover, FDs are excellent vehicles for oral drug delivery as they are safe and allow for the controlled release of the drug at a specific site with a limited amount of aqueous medium, such as the buccal cavity. Additionally, they recommend studying the drug release profile of FDs in the simulated liquid at the application site, such as an artificial salivary solution. Further, they investigated the disintegration profiles of FDs prepared using pectin by measuring the amount of pectin dissolved from the films in a finite amount of aqueous medium. The study examined the relationship between the rate of decay of FDs and the rate of drug release, using miconazole and dexamethasone as standard drugs. The FDs gradually broke down when exposed to water, and the disintegration rate varied depending on the type of pectin used. The speed at which the drug was released from the FDs was also influenced by the dissolution rate of the film matrix. The findings indicate that FDs made with pectin have the advantage of high solubility in a limited amount of medium. The rate of drug release can be controlled by selecting a specific type of pectin or by adjusting the concentration of the film basis [130].

New biopolymer films were developed by modifying bacterial cellulose (BC) with highly methoxylated pectin (HMP) for drug delivery purposes. The films were tested for their ability to incorporate an antibiotic, levofloxacin (Levo). Characterization studies showed that both polymers worked together to produce a strong network, causing significant structural changes in the BC matrix. HMP also reduced water loss in the BC films from 93% to 75% after 90 min. The film ability to absorb macromolecules was tested using human serum albumin (HSA) as a model protein. The presence of HMP increased the encapsulation efficiency of HSA by over 3.5 times, and the release kinetics of both molecules showed sustained-release hyperbolic profiles. When HMP was incorporated into the BC-HMP matrix, the release rates of both macromolecules decreased by about 50%. The incorporation of HSA into the BC-HMP matrix also affected the release profile of Levo. The BC-HMP-HSA films containing Levo were found to have antimicrobial properties against S. aureus. Tests conducted in a laboratory setting showed that the compounds released from the films did not have any negative effects on CHO mammalian cells [131].

The aim of this study was to assess the distribution and behavior of pectin/ethylcellulose film-coated and uncoated pellets containing 5-fluorouracil (5-FU) in rats. Both coated and uncoated pellets were administered orally to rats at a dose of 15 mg/kg. Using high-performance liquid chromatography (HPLC), the concentration of 5-FU in various segments of the gastrointestinal (GI) tract and plasma were quantitatively analyzed. Uncoated pellets released 5-FU primarily in the upper GI tract, while coated pellets released 5-FU in the cecum and colon. This provides an opportunity to improve antitumor efficacy with low systemic toxicity for the treatment of colon cancer through the relatively high local drug concentration and prolonged exposure time [132].

A recent study has shown that pectin combined with calcium or chitosan can effectively slow down drug release, making it a potential option for colon-specific drug delivery. In comparison to uncoated pellets, both calcium-coated and chitosan-coated pellets displayed reduced drug release in 0.1M HCl (1 h) and phosphate buffer with a pH of 6.8 (4 h). The most effective combination resulted in only a 17% drug release during the test period, while uncoated pellets had a drug release of 80%. Results indicated that chitosan was more effective than calcium as a crosslinking agent in reducing drug release. Furthermore, pectin with a degree of methoxylation (DM) of 35 was found to be superior to pectin with DM 17 when combined with chitosan. The drug release rate was further slowed down by using a chitosan type with a high degree of deacetylation (Dda) of 89% and coating at low concentrations (0.1%) in the immersion solution [133].

## 4. Important Methods in Polysaccharide-Based Coatings Characterization

In order to collect adequate data to enable the full characterization of the studied polysaccharides, a broad range of analytical methods were required. These methods are highlighted below. Thin films have unique physical and chemical properties compared to their bulk counterparts because of the geometric limitations they face. Whether in the form of polycrystalline, single crystal (epitaxial), or amorphous, the large surface-to-volume ratio of thin films results in higher surface energy, which accounts for their distinct material and chemical characteristics [134].

Polysaccharides are composed of multiple components, have intricate structures, and possess high molecular weights. Therefore, they are typically studied by analyzing from various perspectives:

(i) Sugar content identification. A developer–sulfuric acid method is often used to measure the sugar content in the sample. When sulfuric acid is used to break down monosaccharides, polysaccharides, and their derivatives, they are converted into aldehyde derivatives, which can then be combined with phenols or aromatic amines to generate colored compounds. By measuring the intensity of these colors, the amount of polysaccharides present can be estimated. These colorimetric methods are efficient, precise, and reliable, with the added benefit of stable coloration [135].
(ii) Molecular weight evaluation. Measuring the molecular weight of polysaccharides is challenging, and there is no single absolute method. Instead, the statistical average method is typically used. In the past, methods such as osmotic pressure, terminal group analysis, viscosity measurement, and light scattering were commonly used, but they were complex and prone to errors. At present, the most commonly used methods are gel filtration and high-performance liquid chromatography, which require standard polysaccharides of known molecular weight as a reference. Mass spectrometry can be used for polysaccharides with a molecular weight of less than 50,000 [136].
(iii) Component identification. There are three main methods for analyzing polysaccharide composition: chemical, physical (instrumental), and biological. Chemical analysis involves acid hydrolysis, neutralization, and filtration. Following this, paper chromatography (PC), thin layer chromatography (TLC), gas chromatography (GC), liquid chromatography (HPLC), or ion chromatography are used for analysis. Some widely used instrumental analyses include spectrophotometry, infrared spectroscopy, nuclear magnetic resonance, and mass spectrometry (MS) [137].
(iv) Structure investigations. Compared to proteins, polysaccharides have more intricate macromolecular structures. Identifying these structures can be complicated due to the diversity of monosaccharides, linking methods, and the complexity of the branching chains. Currently, the primary focus of polysaccharide structure determination is analyzing the molecular weight range of polysaccharides, the type, proportion, and linking order of monosaccharides, and the configuration of glycosidic bonds. Common methods for structural analysis include periodate oxidation, Smith degradation, and methylation reaction. In recent years, advanced instruments such as ultraviolet have greatly improved analysis [138].

There are various methods used to analyze the structure of polysaccharides. These include high performance gel permeation chromatography (HPGPC), osmotic pressure, light scattering, viscosity, and polypropylene gel electrophoresis. These methods provide an overall structural analysis, as well as information about homogeneity and molecular weight.

Other methods, such as complete acid hydrolysis, HPLC, GC, GC-MS, and high performance ion chromatography, can give valuable information about the composition and proportion of monosaccharides.

Raman spectroscopy and infrared spectroscopy can reveal the glycoside ring form. Meanwhile, selective acid hydrolysis, sequential hydrolysis of glycosidase, nuclear magnetic resonance, and evidence of the glycosidic linkage sequence can provide further insights into the structure of polysaccharides [139].

There are various methods available for identifying hydroxyl substitution, such as methylation, periodate oxidation, Smith degradation, GC-MS, and nuclear magnetic resonance. Polysaccharide chain–peptide bond linkage analysis can be carried out using dilute alkali hydrolysis, hydrazine reaction, and amino acid composition reaction methods. For amorphic form substituted by glycosides, glycosidase hydrolysis, nuclear magnetic resonance, infrared chromatography, and laser techniques can be useful. Monosaccharide residue type and glycosidic linkage site can be determined using methylation analysis and GC-MS methods. Oligosaccharide determination analysis can be conducted using partial acid hydrolysis, GC-MS, and MS techniques [140].

Below, Table 6 highlights the importance of the chromatographic method in detecting homogeneity, determining molecular weight, and analyzing monosaccharide composition.

## 5. Challenges towards Commercialization of Polysaccharide-Based Drug Delivery Systems

Despite the great interest in polysaccharide-based therapeutics and the fact that most of the technologies studied have demonstrated their efficacy in vitro and/or in vivo, only a few of these products have made it to the market because of clinical, industrial, economic, and regulatory obstacles [149].

In terms of market share, a few major players dominate the medical products’ market such as Abbott Laboratories, Astra Zeneca, Novartis, Zimmer Biomet, Pedinol, Vertex, CONMED Corporation, Tibotec, Fujisawa, Janssen, Stryker Corporation (Table 7).

In order to advance research and development in the field of polysaccharide-based coatings, it is crucial to conduct a comprehensive review of key marketed patents and technologies. By analyzing these patents, researchers can gain insights into the commercialization potential of these coatings in various industries and identify new coating formulations, fabrication techniques, and methods for enhancing the stability and functionality of these coatings. Understanding the latest developments can guide researchers and developers in designing coatings that meet specific market needs, leading to successful commercialization. Next, in order to analyze the commercial success of existing products and identify gaps or opportunities in the market, we have centralized some of the available key marketed patents or technologies in Table 8.

Challenges towards the commercialization of polysaccharide-based coatings from technological perspectives involve several factors that need to be considered. A list of concrete challenges and indications for both pristine and polysaccharide-based coatings is detailed as follows:

(i) Polysaccharides are often heterogeneous, meaning that they can have variations in their repeating units and structures. This complexity poses challenges in terms of characterization, standardization, and quality control. The variations in polysaccharide structures can affect their functional properties and, of course, the coating characteristic, making it necessary to develop reliable and efficient methods to analyze and characterize either pristine and polysaccharide-based coatings [163].
(ii) Even the fact that polysaccharides are renewable resources (being typically extracted from natural sources such as plants, algae, and microorganisms) represents a big plus, the extraction process can be complex and may require optimization in order to ensure high yields and purity of polysaccharides. Additionally, purification steps may be necessary to remove impurities and contaminants [164]. Developing efficient and cost-effective extraction and purification methods is crucial for the commercial production of polysaccharides and polysaccharide-based coatings.
(iii) Scaling up the production of polysaccharides from laboratory to industrial levels can be challenging. Factors such as yield, cost, and scalability need to be considered. The production processes should be optimized to ensure consistent quality, high yields, and affordable cost [163]. Strategies such as fermentation, enzymatic synthesis, and biotechnology approaches may be employed to enhance polysaccharide production [163].
(iv) Polysaccharides may exhibit different properties depending on their structures and interactions with other components. Formulating polysaccharides into stable products such as gels, films, or coatings, requires accurate understanding their physicochemical properties and optimizing the formulation parameters [163]. Stability during storage, transportation, and use, is also a critical factor to consider for commercialization.
(v) Polysaccharides intended for commercial use may need to comply with regulatory requirements and safety standards. These requirements may vary, depending on the intended application and region in which the polysaccharides will be used. Safety assessments, including toxicity and allergenicity studies, may be necessary to ensure the safety of polysaccharide-based products [163].
(vi) The commercial success of polysaccharides depends on market acceptance and cost considerations. Polysaccharides should offer unique functional properties and advantages compared to existing alternatives. The cost of production, including raw materials, extraction, purification, and formulation, should be competitive in the market [165].

To summarize, some polysaccharides, such as beta-glucan from different fungi, have undergone clinical trials as immunomodulators and anticancer agents and have achieved commercial success in certain countries [166]. However, there is still a significant obstacle in the development of polysaccharide-based coatings for controlled drug release.

When considering commercialization, it is important to take into account some obstacles such as the limitations of the characterization techniques, high costs of purification, as well as the heterogeneity and diversity of natural polysaccharides [167]. In the commercial production of products, there is a risk of inconsistent structural attributes between batches, such as differences in monosaccharide composition, molecular weight, size, degree of branching, linkage, and more. This variability is undesirable [168].

Currently, some studies related to the polysaccharide-based transdermal drug delivery vehicles, such as hydrogel, film, microneedle, and tissue scaffolds, are exploring ways to reduce the risk of medical device infection during surgery [8]. One area of focus is the use of coatings and metals with antibacterial properties. However, it is not yet clear if these coatings can effectively prevent infection in the early days after the device is implanted. More research is needed in this area [169].

Medical product research requires the use of animal models that accurately mimic human conditions to better understand the complex process of osseointegration. Before new biomedical product designs can enter the market, extensive preclinical and clinical studies are necessary, with a focus on long-term functional studies [170]. Only after addressing these limitations, undergoing rigorous quality control, and adhering to regulations can polysaccharide-based therapeutics achieve commercial success.

In conclusion, the commercialization of polysaccharides faces challenges related to their complexity, extraction, purification, scaleup, formulation, stability, regulatory compliance, and market acceptance. Overcoming these challenges requires interdisciplinary collaboration, technological advancements, and optimization of production processes. Further research and development efforts are needed to address these challenges and unlock the full potential of polysaccharides in various industries.

## 6. Conclusions and Future Perspectives

Polysaccharide-based coatings for drug delivery systems have enormous potential because of their low cost, ease of processing, biocompatibility, and biodegradability. In biotechnology, the current focus for medical devices centers on fabricating functionalized surfaces and bioactive polysaccharide coatings, providing controlled release and targeted delivery. This is coupled with in situ drug delivery strategies to reduce infection rates and enhance clinical outcomes. Polysaccharide coatings offer various approaches for the administration of drugs in drug delivery systems. These coatings provide controlled release, tissue-specific targeting, stability enhancement, and the ability to overcome physiological barriers. By combining polysaccharide coatings with other delivery systems, the overall performance and functionality of the drug delivery system can be further enhanced. A key point regarding the approaches for the administration of polysaccharides in the form of coatings for drug delivery is related to the fact that these coatings can be applied to drug-loaded particles or implants to protect the drug and control its release over time. The biodegradability of polysaccharides ensures that the coating will gradually break down, releasing the drug in a controlled manner. Polysaccharide coatings can be designed to modulate the release of drugs, allowing for sustained and controlled delivery. The rate of drug release can be adjusted by modifying the thickness or composition of the coating. By controlling the release kinetics, polysaccharide coatings can optimize the therapeutic effect of the drug and minimize side effects. Moreover, polysaccharides have extensive and varied tissue-specific targeting capabilities. By incorporating targeting ligands onto the polysaccharide coating, drugs can be specifically delivered to the desired tissues or cells. This targeted delivery approach enhances drug efficacy and reduces off-target effects, improving the overall therapeutic outcome. Moreover, polysaccharide coatings can stabilize labile therapeutics, protecting them from degradation and improving their shelf life. The coating acts as a barrier, preventing exposure of the drug to external factors that may compromise its stability. This is particularly important for drugs that are sensitive to light, heat, or moisture. Polysaccharide coatings can help to overcome physical and physiological barriers to drug delivery. These coatings can protect the drug from enzymatic degradation in the gastrointestinal tract when orally administered. They can also enhance drug penetration through biological barriers, such as the blood–brain barrier or mucosal barriers, by modulating the surface properties of the drug delivery system. Polysaccharide coatings can be combined with other drug delivery systems, such as nanoparticles or microparticles, to enhance their properties and functionality. This combination allows for synergistic effects, such as improved stability, controlled release, and targeted delivery. The coating can provide an additional layer of protection and enhance the overall performance of the drug delivery system.

Despite these efforts, studies still need to investigate the constraints of thin films, including inadequate drug loading capacity for highly potent drugs administered at elevated doses and their hygroscopic properties that demand careful preservation.

Also, the biointegration still remains a challenge. Hence, developing new methods to enhance the osseointegration of medical coated devices, including novel macroscopic designs and surface modifications, is crucial. Further research is required to enhance the performance of polysaccharide-based coatings, with a focus on improving the bond strength and interface between the coating and surface of medical devices. By manipulating the composition and fabrication methods, polysaccharide-based thin films can be designed to meet specific drug delivery requirements for various applications. It is important to prioritize the development and implementation of specialized coatings that are customized for each patient, as this will ensure the continued success of current commercial products in the long term.

## Figures and Tables

**Table 1:** Advantages and disadvantages of polysaccharide-based coatings as drug delivery systems.

Deposition Technique		Advantages	Disadvantages
Vapor Deposition Techniques	Physical Ex.: matrix-assisted pulsed laser evaporation (MAPLE)	Applied to both organic and inorganic coatings, multilayers and multi structures, nanoparticulate films, and thickness control [23].	Generation of micrometer-sized droplets and particulates on surfaces, small covering areas [24].
	Chemical	Very high deposition rate and can produce thick coatings [25].	It required a very high temperature [26].
Interfacial Deposition Processes	Colloidal Solutions Ex.: sol–gel	Available for both organic and inorganic coatings, simple operation, high versatility [26].	Poor adhesion to substrates, difficulty in producing multilayers due to solvent issues, and difficulty in generating gradient coatings [27].
	Langmuir-Blodgett	Possibility to assemble monolayers, high spatial coverage [28].	Limited to very thin films, typically used for organic coatings only [28].
Other Deposition Techniques	Ex.: atomic layer deposition	High-quality films could be produced at low temperature [29].	Very high energy waste rate [29].

**Table 2:** An overview of drug delivery systems based on polysaccharides.

Polysaccharide	Formulation and/or Administration Route of Delivery System. Stage of Development	Drug	References
Starch	Coatings: oral drug delivery systems for in vitro drug release assays.	Bovine Serum Albumin	[49]
	Hydrophobic modification attaches and stabilizes hydrophobic drugs (transdermal drug delivery systems based on nanoparticles for in vitro drug release studies).	TestosteroneCaffeine Flufenamic acid	[50]
Cationic Chitosan	Coatings: on metallic implants for in vitro drug release tests.	Vancomycin or daptomycin	[51]
	Mucoadhesive nanocarriers for in vivo intestinal adhesion and permeation study. Polyelectrolyte hydrogel. Charge tailored to the active substance binding. Stabilization of labile active substances in gastric disorders. Chitosan provides mucosal binding and transmucosal transport.	DNA/RNA Therapeutic peptides and proteins Doxorubicin	[52]
	Nanogels for cytotoxicity evaluations. Ionic crosslinking ammonium groups promote transmucosal delivery.	Model macromolecule therapeutics Furosemide Doxorubicin Triclosan	[53]
	Colloid systems based on chitosan for physicochemical characterization. Adjustable size drug release particles.		[54]
Neutral Dextran	Nanoparticles for gene delivery; in vivo studies. This has been adapted for use with polyelectrolyte hydrogel. The tuning of the charge for drug binding has been optimized.	Therapeutic peptides and proteins Doxorubicin	[55]
	Coatings: on percutaneous implants, in vitro studies.	Minocycline	[56]
	Nanoparticle polysaccharide–drug conjugates for in vivo antitumor efficacy, modified for amphiphilic self-assembly. Covalently bound drugs.	Curcumin DoxorubicinBortezomib	[57]
Anionic Hyaluronic Acid	Coatings: on implant coating; in vitro tests.	Vancomycin or tobramycin	[58]
	Different formulations as nanoparticles, micelles, nanogels, liposomes, and nanocapsules have been used as vehicles, following various administration routes such as parenteral, ocular, dermal, nasal, and oral. In vitro and in vivo studies. Amphiphilic self-assembly. Target cancer cells.	Salinomycin EtoposideDoxorubicinCurcuminPaclitaxel	[59]
	Injection for in vitro and in vivo signal intensity induced by GCHN. Polyelectrolyte hydrogel. Target cancer cells	MicroRNA Doxorubicin	[60]
	Biodegradable hydrogels for cytocompatibility study. Covalently crosslinked biodegradable hydrogel.	Therapeutic peptides and proteins	[61]
Arabinogalactan	Coatings: coating of cisplatin with polysaccharides and specific carriers for targeted delivery for in vivo antitumor effect.	Cisplatin	[62]
	Drug delivery systems with hepatocyte asialoglycoprotein receptor binding targeting the liver for in vivo antitumor efficacy. Self-assembled particles. Binds asialoglycoprotein receptors. Targets liver and cancer tumors.	Norcantharidin	[63]
Neutral Pullulan	Coatings: gellan gum/pullulan bilayer film containing silibinin-loaded nanocapsules for topical treatment of atopic dermatitis; in vivo study.	Silibinin	[64]
	Therapeutic molecules to directly target various body organs such as liver, lungs, brain, spleen; proficient gene carrier for in vitro studies. Self-assembly of amphiphiles can bind and safeguard hydrophobic drugs and dyes.	Model protein IRDye 800	[65]
	Hydrodynamics- or nonhydrodynamics-based injection for in vivo studies. PEI modification assembles into particles. Targets asialoglycoprotein receptors in the liver	siRNA	[66]
Anionic GumsPectin	Polysaccharide-based theranostic systems—nanoparticles—for in vivo studies Delivery of substances orally to the colon. Degradation caused by gut bacteria.	Protein and polypeptide drugs	[67]
	Coatings: buccal films; mucosal delivery of drugs for in vitro drug release studies.	Clotrimazole	[68]

**Table 3:** Starch-based coatings as drug delivery systems: advantages vs. disadvantages.

Polysaccharide Coatings as Drug Delivery Systems	Advantages	Disadvantages
Starch-Based Coatings as Drug Delivery Systems	Natural, renewable available, nontoxic, biodegradable, low cost, biocompatibility, tailorable characteristics [69].	Hydrophilicity, fragility, high viscosity, poor mechanical properties, poor resistance to external factors [70].

**Table 4:** Chitosan-based coatings as drug delivery systems: advantages vs. disadvantages.

Polysaccharide Coatings as Drug Delivery Systems	Advantages	Disadvantages
Chitosan-Based Coatings as Drug Delivery Systems	Less toxicity for drug administration, enhanced biocompatibility, process stability, site-specific drug targeting, possess mucoadhesive character, therapeutic index of the drug is increased [84].	Less mechanical resistance, difficulty in controlling pore size, may contract, low solubility in neutral and alkaline pH, preparation by crosslinking can affect intrinsic properties of chitosan [84].

**Table 5:** Dextran-based coatings as drug delivery systems: advantages vs. disadvantages.

Polysaccharide Coatings as Drug Delivery Systems	Advantages	Disadvantages
Dextran-Based Coatings as Drug Delivery Systems	Low cost, biodegradability, biocompatibility, nontoxicity, decreased blood viscosity, decreased platelet adhesiveness [92].	Briefer volume expansion; the highest incidence of anaphylactic reactions; interferes with blood grouping, clotting; antiplatelet; worsens renal failure; hyperviscosity syndrome in renal tubules [92].

**Table 6:** Structural analysis of polysaccharides.

Identification of Primary Structure of Polysaccharides	Principle of the Method
Detection of polysaccharide homogeneity	Chromatography An example could be gel permeation chromatography and HPLC in combination with a differential refractive index detection (RID). To conduct HPLC, the polysaccharide is initially broken down into monosaccharides and chemically modified, such as by adding a fluorescent group to enhance detection sensitivity. This is achieved through the use of a derivative reagent, with 1-phenyl-3-methyl-5-pyrazolone (PMP) being a commonly utilized option [141]. Gel permeation chromatography is a method that can be used to determine the molecular weight of a polysaccharide [142]. In the gel column, polysaccharides with varying masses can move at varying speeds [142]. Therefore, an appropriate flow rate can be used to elute different components of the polysaccharide sample. The absorbance curve is plotted using the tube number and absorbance measurement as the ordinates. When a single and symmetrical peak appears using this method, the component is typically considered to be a homogeneous polysaccharide [140]. One way to measure the amount of polysaccharides present is through the use of the phenol–sulfuric acid method [140].
	Other Methods: Polyacrylamide gel electrophoresis and cellulose acetate membrane electrophoresis are used in conjunction with GPC to confirm the purity of polysaccharides and determine their molecular weight [143].
Determination of molecular weight of polysaccharides	High Performance Liquid Chromatography Some examples of chromatography techniques that offer advantages such as speed, high resolution, and reproducibility while detecting polysaccharide homogeneity are high-performance gel permeation chromatography (HPGPC) and high-performance exclusion chromatography (HPSEC) [144]. Some commonly used column types for scientific analysis are μ-Bondagel, TSK, Sephadex, and Superose. The mobile phases used in these columns include water, buffer, or aqueous organic solvent. Various detectors are also used such as refractive index refractometers, evaporative light scattering detectors, and multiangle excitation diffusers [145].
	Gel Permeation Chromatography In certain practical applications, GPC is utilized in conjunction with a multiangle laser scattering detector (MALLS) [146]. This technique boasts high accuracy and precision, without the need for calibration, using a reference material.
	Mass Spectrometry MALDI-TOF-MS is a common tool for analyzing biological macromolecules. Various techniques, including collision-induced cleavage, electron transfer cleavage, electron capture cleavage, post-source decay, and other post-source cleavage methods are utilized to determine the molecular weight of polysaccharides and identify structural fragments [147]. To achieve the desired outcome, the concentration of the sample and the matrix are chosen based on the structure of the polysaccharide during the measurement process.
Determination of monosaccharide composition	High-Performance Liquid Phase Chromatography (HPLC)
	High-Performance Capillary Electrophoresis (HPCE) To analyze the polysaccharide, it is first labeled with an acidic reagent, and then its composition is detected using a laser-induced fluorescence detector. It has been found that polysaccharides can be broken down into monosaccharides through acid hydrolysis and then derivatized using PMP [140]. The sample is then examined by the device. The mole percentage of monosaccharides can be calculated from the peak area [148].
	Ion Chromatography By using high-performance anion exchange chromatography in conjunction with pulsed amperometric detection (HPAEC-PAD), it is possible to analyze hydrolytic products directly without needing to derive samples [140]. When hydrolyzed polysaccharides are treated with a pH > 12 elution solution, the resulting monosaccharides and oligosaccharides can be separated into anions. This process involves exchanging and distributing the polysaccharides on a high-efficiency anion exchange resin and detecting them with a pulsed amperometric detector. The device used for this purpose contains an electrochemical detector that has a Au working electrode and a Ag reference electrode. A step-gradient elution is performed using a solution of pure water, sodium hydroxide, and sodium acetate. Comparing the monosaccharide composition with standard samples such as glucose, fructose, galactose, and mannose helps to determine the composition [135].
	Formation Of Methyl Glycosides When analyzing the monosaccharide composition with GC-MS, it is important to measure the content of uronic acid separately. Uronic acid is present in polysaccharide structures and cannot be analyzed as part of the monosaccharide analysis [135,140].

**Table 7:** Polysaccharide-based marketed products [150].

Marketed Product	Drug	Polysaccharide Carrier	Description	Reference
NOXAFIL (manufacturer: Merch Sharp & Dohme Ltd.; year of approval: 2014)	Posaconazole	Hypromellose acetate succinate (HPMCAS)	A tablet with a delayed release that resists gastric acid was produced. It contains a substance that restricts posaconazole release when pH is low but releases it when pH is neutral.	[151]
KALYDECO (manufacturer: Abbott Laboratories; year of approval: 2012)	Ivacaftor	Hypromellose acetate succinate (HPMCAS)	Indication for cystic fibrosis How it works? Cystic fibrosis is caused by changes in the CFTR gene, which affects the production of the CFTR protein that controls mucus and digestive juices. Kalydeco, containing ivacaftor, helps to alleviate symptoms by boosting the protein’s activity.	[152]
CRESTOR (manufacturer: Astra Zeneca; year of approval: 2003)	Rosuvastatin	Hydroxypropyl methylcellulose (HPMC)	Indication for use as a hypolipidemic agent How it works? Crestor, also known as rosuvastatin calcium, is a type of medication called a selective 3-hydroxy-3-methylglutaryl coenzyme A (HMG-CoA) reductase inhibitor or statin. It has been approved for managing dyslipidemia, which is a condition characterized by abnormal levels of lipids in the blood.	[153]
Votubia (manufacturer: Novartis; year of approval: 2015)	Everolimus	Hydroxypropyl methylcellulose	Indication for treatment of benign (noncancerous) tumors caused by the genetic disease tuberous sclerosis. How it works? Votubia is an antitumor drug that blocks an enzyme called mTOR, which is more active in tumor cells of patients with SEGA or renal angiomyolipoma. It inhibits tumor cell division and reduces their blood supply by targeting cell division control and the growth of blood vessels. It is not fully understood how Votubia works to prevent seizures in patients with tuberous sclerosis, but mTOR is believed to play a role.	[154]
Zepatier (manufacturer: Merck; year of approval: 2016)	Elbasvir/Grazoprevir	HPMC, D-α-tocopheryl polyethylene glycol succinate (TPGS), and copovidone	This treatment is intended for adults and children aged 12 and above, who weigh at least 30 kg, and are suffering from chronic hepatitis C. This is an infectious disease that affects the liver and is caused by the hepatitis C virus. How it works? Zepatier contains elbasvir and grazoprevir, which are active substances that prevent hepatitis C virus from multiplying. Elbasvir targets the protein NS5A, while grazoprevir targets the enzyme NS3/4A protease. By blocking the action of these proteins and enzymes, Zepatier effectively stops the virus from infecting new cells.	[155]
Envarsus (manufacturer: Veloxis Pharmaceuticals; year of approval: 2015)	Tacrolimus	Poloxamer/HPMC	Indication for prevention of organ rejection in adult patients who have undergone kidney or liver transplants. It can also be used to treat rejection when other medications have failed. How it works? Envarsus is an immunosuppressive medicine that decreases the activity of T cells in the immune system to prevent organ rejection.	[156]

**Table 8:** Marketed patents/technologies [157].

Marketed Patent/Technology	Polysaccharide-Based Drug Delivery Systems	Reference
Water insoluble polymer: indigestible water-soluble polysaccharide film coatings for colon targeting. Patent no. US9107819B2. 18 August 2015	This formulation contains prednisolone sodium metasulfobenzoate, which is surrounded by a coating made up of glassy amylose, ethyl cellulose, and dibutyl sebacate. The ratio of amylose to ethyl cellulose falls between 1:3.5 and 1:4.5, and the amylose used is derived from either corn or maize.	[158]
Hyaluronic acid modification products and drug carrier using them. Patent no. US7767806B2 3 August 2010	This product is a modified version of hyaluronic acid that includes a polymer bonded to it. The polymer can be polylactic acid, polyglycolic acid, or lactic acid–glycolic acid copolymer. This modified hyaluronic acid serves as an efficient carrier for low molecular weight drugs, providing sustained-release over a long period of time, controlling blood residence, and being well-dispersible in an aqueous solution. Moreover, it has excellent biocompatibility.	[159]
Tsiros, D. and Nugent, M.A. Compositions and methods for the treatment of angiogenesis-related diseases (2020). Patent no. WO2020219497A1 29 October 2020	Avastin, also known as Bevacizumab, is an antibody that works by binding to VEGF and preventing it from binding to VEGF receptors on endothelial cells. It is commonly used to treat cancers and other diseases that involve excessive growth of blood vessels. To enhance its anti-VEGF activity, Avastin was conjugated with either biotin or streptavidin, and biotin–heparin was utilized to bring the two molecules closer together through biotin–streptavidin binding.	[160]
Drug-coated medical devices. Patent no. CN107206129B 2 March 2021	A drug-coated balloon is designed to enhance the efficiency of drug transfer and reduce drug loss. These balloons are primarily made up of a single active pharmaceutical layer that uses small, hydrophilic molecules as carriers. Examples of these carriers include urea, sorbitol, polysorbate, micelles, oil or lipid vehicles, contrast agents, iopromide, iodophorol, resveratrol, surfactants, and sodium alginate polysaccharide. It is important to note that increasing the efficiency of drug transfer can sometimes lead to a higher amount of drug loss, and vice versa.	[161]
Medical implants with polysaccharide drug-eluting coatings Patent number: US7939096B2 10 May 2011 Publication of US7939096B2	A medical implant has a bioerodible metal section and a protective layer. The layer has a therapeutic substance and a polysaccharide matrix, cross-linked with metal cations. Upon insertion, the substance is released, and the metal dissolves to recrosslink the matrix.	[162]

## Data Availability

The data presented in this study are available on request from the corresponding author.

## Abbreviations

1,4-Butanediol diglycidyl ether	(Ti-HABDDE)
1-Phenyl-3-methyl-5-pyrazolone	(PMP)
2,4-Dinitrochlorobenzene	(DNCB)
2-Chloro-N,N-diethylethylamine	(DE-AE)
3-Hydroxy-3-methylglutaryl coenzyme A	(HMG-CoA)
45S5 Bioglass	(BG)
5-Fluorouracil	(5-FU)
Acetylsalicylic acid (aspirin)	(ASA)
Adamantane-modified hyaluronic acid	(HAD)
Alpha-smooth muscle actin, commonly known as actin alpha 2, Acta2	α-SMA
Aptamer	AS-42
Arabinogalactan	(AG)
Asialoglycoprotein receptor	(ASGPR-1)
Atomic force microscopy	(AFM)
Atopic dermatitis	(AD)
Bacterial cellulose	(BC)
Beta-cyclodextrin-modified silk fibroin	(SCD)
Bone morphogenetic protein 2	BMP2
Cancer drug methotrexate	(MTX)
Carbon tetrachloride	(CCl4)
Carboxymethyl starch	(CMS)
Carboxymethyl pullulan	(CMP)
Carboxymethyl dextran	(CMD)
Cefazolin/chitosan	(P-Z@C)
Cefazolin/chitosan crosslinked by calcium phosphate	(P-Z@C/CP)
Chitosan/carboxymethyl pullulan	(CCMP)
Chitosan/carboxymethyl pullulan polyelectrolyte complex	(PEC)
Chitosan/carboxymethyl pullulan polyelectrolyte complex (PEC) loaded with 45S5 Bioglass	(CCMPBG)
Cisplatin–arabinogalactan–aptamer	(Cis-AG-Ap)
Collagen-1-alpha	COL1A1
Corn starch acetates	(SA)
Crosslinked MC-loaded nanocells	(MC@(ODA-CMD)CL
DEAE-modified pullulan	(PULL-DEAE)
Defensive Antibacterial Coating	DAC^®^
Degree of methoxylation	(DM)
Dextran-coated cerium-doped hydroxyapatite (Ca_10−x_Ce_x_(PO_4_)_6_(OH)_2_) with x = 0.05	(5CeHAp-D)
Dextran-coated cerium-doped hydroxyapatite (Ca_10-−x_Ce_x_(PO_4_)_6_(OH)_2_) with x = 0.1	(10CeHAp-D)
Differential refractive index detector	(RID)
Divinyl sulfone	(Ti-HADVS)
Dosage forms	(FDs)
Drug Delivery System	(DDS)
Dynamic light scattering	(DLS)
D-α-tocopheryl polyethylene glycol succinate	(TPGS)
Encapsulation efficiency	(EE)
Endosomal cleavable peptide	(GFLG)
Energy-dispersive X-ray spectroscopy	(EDX)
Essential oil	(EO)
Fibrosis-mediating proteins	PDGFRB, TIMP-1, TLR-9 and TGFβ
Folate receptors	(FR)
Folic acid	(FA)
Fourier transform infrared spectroscopy	(FTIR)
Gas chromatography	(GC)
Gas chromatography–mass spectrometry	GC-MS
Gastrointestinal	(GI)
Gel permeation chromatography	GPC
Glow discharge optical emission spectroscopy	(GDOES)
Grafted copolymer pullulan–DEAE-g-poly(Z-1-lysine)	(PULL–DEAE-g-PZLL)
Heat shock proteins	(HSP)
High-performance liquid chromatography	(HPLC)
Highly methoxylated pectin	(HMP)
High-performance anion exchange chromatography combined with pulsed amperometric detection	(HPAEC-PAD)
High-performance capillary electrophoresis	(HPCE)
High-performance exclusion chromatography	(HPSEC)
High-performance gel permeation chromatography	(HPGPC)
Human liver cancer cell line	(HepG2)
Human serum albumin	(HSA)
Hyaluronic acid	(HA)
Hydroxypropyl methylcellulose	(HPMC)
Hypromellose Acetate Succinate	(HPMCAS)
Ibuprofen	(IBU)
Lactobionic acid	(LA)
Lactobionic acid–adipic acid dihydrazide conjugate	(LAD)
Lauroylated AG	(LAG)
Lauroylated PL	(LPL)
Layer-by-layer	(LbL)
Levofloxacin	(Levo)
Local drug delivery	(LDD)
Mammalian cells	CHO
Mass spectrometry	(MS)
Matrix-assisted pulsed laser evaporation	(MAPLE)
MC-loaded CMD-based nanocells coated on a MAO-Ti surface	(MC@(ODA-CMD)CL-Ti)
Metallographic microscopy	(MM)
Microarcoxidized titanium	(MAO-Ti)
Minimum inhibitory concentration	(MIC)
Minocycline	(MC)
Molecular weights	(MW)
Multiangle laser scattering detector	(MALLS)
Nanocomposites	(NCs)
Nanoparticles	(NPs)
Nanostructured lipid carriers	(NLCs)
N-carbobenzyloxy-1-lysine-N-carboxyanhydride	(Lys(Z)-NCA)
Nuclear magnetic resonance	(NMR)
Octadecylamine	(ODA)
Palmitoylated PL	(PPL)
Paper chromatography	(PC)
Platelet-derived growth factor receptor beta	PDGFRB
Platelet-derived growth factor receptor beta	(PDGFRB)
Poly(glycerol adipate)	(PGA)
Poly(methyl methacrylate)	(PMMA)
Polyamide 12	(PA12)
Polycaprolactone	(PCL)
Polysaccharide arabinogalactan	(AG)
Pomegranate peel extract	(PPE)
Porous starch	(PS)
Pullulan	(PL)
Pullulan–polyethyleneimine	(Pul-PEI)
Pullulan–tris(2-aminoethyl)amine	(Pul-TAEA)
Pulsed direct current	(DC)
Quercetin	(Quer)
Salicylic acid	(SA)
Scanning electron microscopy	(SEM)
Silver nanoparticles	(AgNPs)
*Staphylococcus aureus*	(*S. aureus*)
The most biologically relevant receptor	(CD44)
The receptor for hyaluronan-mediated motility	(RHAMM)
Thin-layer chromatography	(TLC)
Tissue inhibitors of metalloproteinases	(TIMPs)
Transmission electron microscopy	(TEM)
Triblock copolymer	(Pluronic F127)
Palmitoylated AG	(PAG)
